# Characterization of a sweet basil acyltransferase involved in eugenol biosynthesis

**DOI:** 10.1093/jxb/eraa142

**Published:** 2020-03-21

**Authors:** Niha Dhar, Sreelatha Sarangapani, Vaishnavi Amarr Reddy, Nadimuthu Kumar, Deepa Panicker, Jingjing Jin, Nam-Hai Chua, Rajani Sarojam

**Affiliations:** 1 Temasek Life Sciences Laboratory, 1 Research Link, National University of Singapore, Singapore; 2 Laboratory of Plant Molecular Biology, The Rockefeller University, New York, NY, USA; 3 Singapore Centre on Environmental Life Sciences Engineering, Nanyang Technological University, Singapore; 4 China Tobacco Gene Research Centre, Zhengzhou Tobacco Research Institute of CNTC, Zhengzhou, China; 5 Chinese Academy of Sciences, China

**Keywords:** BAHD acyltransferase, coniferyl alcohol, eugenol, lignin, phenylpropene, sweet basil *(Ocimum basilicum)*

## Abstract

Sweet basil (*Ocimum basilicum*) plants produce its characteristic phenylpropene-rich essential oil in specialized structures known as peltate glandular trichomes (PGTs). Eugenol and chavicol are the major phenylpropenes produced by sweet basil varieties whose synthetic pathways are not fully elucidated. Eugenol is derived from coniferyl acetate by a reaction catalysed by eugenol synthase. An acyltransferase is proposed to convert coniferyl alcohol to coniferyl acetate which is the first committed step towards eugenol synthesis. Here, we perform a comparative next-generation transcriptome sequencing of different tissues of sweet basil, namely PGT, leaf, leaf stripped of PGTs (leaf–PGT), and roots, to identify differentially expressed transcripts specific to PGT. From these data, we identified a PGT-enriched BAHD acyltransferase gene *ObCAAT1* and functionally characterized it. *In vitro* coupled reaction of ObCAAT1 with eugenol synthase in the presence of coniferyl alcohol resulted in eugenol production. Analysis of *ObCAAT1*-RNAi transgenic lines showed decreased levels of eugenol and accumulation of coniferyl alcohol and its derivatives. Coniferyl alcohol acts as a common substrate for phenylpropene and lignin biosynthesis. No differences were found in total lignin content of PGTs and leaves of transgenic lines, indicating that phenylpropene biosynthesis is not coupled to lignification in sweet basil.

## Teaser

The sweet basil glandular trichome-enriched BAHD acyltransferase CAAT1 involved in eugenol formation was characterized. RNAi of CAAT1 leads to a decrease in eugenol and accumulation of coniferyl alcohol and its derivatives.

## Introduction

Plants produce and emit a diverse array of volatile organic compounds (VOCs) which play an important role in defence responses and pollinator attraction. They are also responsible for the characteristic aroma and flavours of herbs and spices, since VOCs are the major components of plant essential oils. Phenylpropenes derived from the phenylpropanoid pathway are one of the largest classes of plant VOCs along with terpenes which are widely used in flavour, perfume, and pharmaceutical industries, making them economically significant. Common plant-produced phenylpropenes include the allylphenols, eugenol and chavicol, and the propenylphenols, *t*-anol and isoeugenol (Louie *et al.*, 2007). Eugenol is found in large amounts in clove, cinnamon, and many varieties of basil (*Ocimum basilicum*), and has huge commercial worth, expected to reach US$754.1 million revenue by 2026 (Gang *et al.*, 2001) (https://www.globenewswire.com/news-release/2018/03/07/1417672/0/en/Anti-Microbial-Properties-of-Eugenol-to-drive-the-Global-Eugenol-Market-to-Reach-US-754-1-million-revenue-by-2026-end.html). Given the commercial and ecological importance of phenylpropenes such as eugenol, understanding their biosynthesis pathways to enable strategies to metabolically engineer them are of considerable interest.

Cloves are considered to be the largest producers of eugenol, but they are not amenable to laboratory manipulations and biochemical investigation as clove buds take many years to mature. Sweet basil (*O. basilicum* L.), in contrast, are cultivated perennial plants that produce high levels of phenylpropenes such as eugenol and chavicol, along with mono- and sesquiterpenes such as eucalyptol, linalyl acetate, and α-bergamotene in their essential oils. These are produced in specialized structures called peltate glandular trichomes (PGTs) which are widely found on aerial surfaces of many aromatic plants. Different basil varieties produce characteristic blends of volatile allylphenol compounds, with eugenol and methylchavicol being the most abundant (Gang *et al.*, 2001). A high level of production of such allylphenol compounds and amenability to laboratory manipulation make *O. basilicum* a model organism to investigate the production of phenylpropenes such as eugenol, facilitating metabolic engineering attempts.

**Fig. 1.**
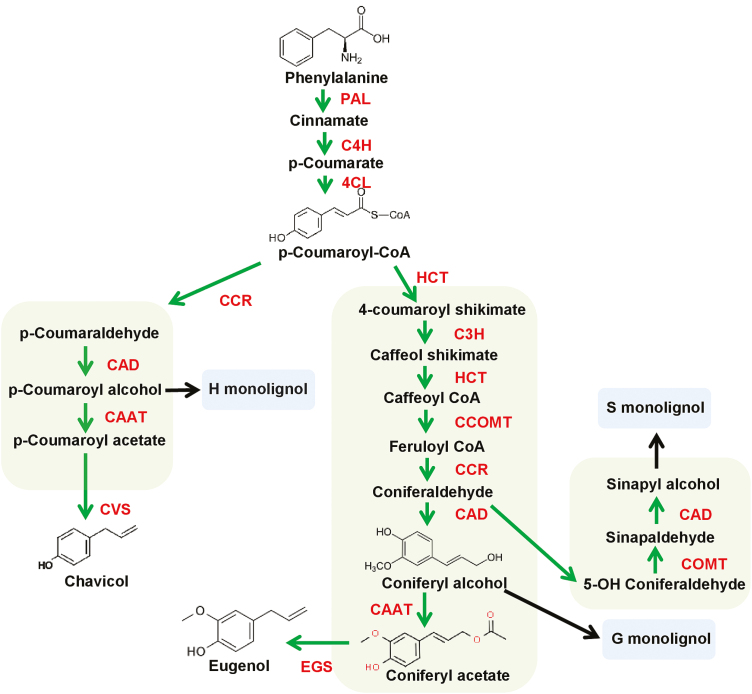
Plant phenylpropanoid pathway. PAL, phenylalanine ammonia lyase; C4H, cinnamate 4-hydroxylase; 4CL, 4-coumaroyl-CoA ligase; CCR, cinnamoyl-CoA reductase; HCT, hydroxycinnamoyl-CoA shikimate/quinate hydroxycinnamoyl transferase; C3H, *p*-coumaroylshikimate 3'-hydroxylase; CAD, cinnamyl alcohol dehydrogenase; CAAT, coniferyl alcohol acetyl transferase; COMT, caffeic acid *O*-methyltransferase; CCOMT, caffeoyl-CoA *O*-methyltransferase; F5H, ferulate-5-hydroxylase; CVS, chavicol synthase; EGS, eugenol synthase.

Phenylpropenes are derived from the general phenylpropanoid pathway beginning from the aromatic amino acid phenylalanine. The three primary steps of the pathway catalysed by phenylalanine ammonia lyase (PAL), cinnamate 4-hydroxylase (C4H), and 4-coumaroyl CoA-ligase (4CL) lead to the formation of 4-coumaroyl-CoA which is the most important branch point metabolite of this pathway (Fig. 1). In addition to phenylpropenes, it serves as a precursor for the synthesis of many compounds such as lignin, flavonoids, anthocyanins, phenolic acids, and stilbenes (Weisshaar and Jenkins, 1998; Vogt, 2010). The phenylpropene and lignin pathway bifurcates after the formation of monolignols, namely coumaryl alcohol, coniferyl alcohol, and sinapyl alcohol (Hamberger *et al.*, 2007). In plants, these monolignols are used as shared intermediates for phenylpropene, lignin, and lignan biosynthesis. Lignin formation involves random polymerization of activated monolignols through ether and carbon–carbon linkages (Ferrer *et al.*, 2008). Lignins polymerized from *p*-coumaryl alcohol, coniferyl alcohol, and sinapyl alcohol are commonly referred to as hydroxyphenyl (H), guaiacyl (G), and syringyl (S) lignin, respectively (Vanholme *et al.*, 2010).

Towards phenylpropene biosynthesis, the monolignols *p*-coumaryl alcohol or coniferyl alcohol are acetylated at the C9 hydroxyl group mediated by acyltransferases to form the acetyl esters *p*-coumaryl acetate and coniferyl acetate, respectively (Dexter *et al.*, 2007).These monolignol acetates undergo a subsequent NADPH-dependent reduction by allylphenol synthase or propenylphenol synthase, to finally form the corresponding allylphenols (chavicol and eugenol) or propenylphenols (*t*-anol and isoeugenol). The plant BAHD acyltransferases are a family of catalytically versatile diverse group of enzymes that transfer an acyl moiety of an acyl-CoA compound to an alcohol to produce a variety of plant secondary metabolites critical to many physiological processes (St-Pierre and De Luca, 2000). The BAHD acyltransferase family was named after the first letter of each of the first four biochemically characterized enzymes of this family [benzylalcohol *O*-acetyl transferase (BEAT), anthocyanin *O*-hydroxycinnamoyl transferase (AHCT), anthranilate *N*-hydroxycinnamoyl/benzoyl transferase (HCBT), and diacetyl vindoline 4-*O*-acetyltransferase (DAT)]. BAHD acyltransferases can acylate various substrates, but the catalytic flexibility of individual enzymes differs greatly (D’Auria, 2006). Most functionally characterized BAHD acyltransferases contain two conserved domains, the catalytic HXXXD domain and a DFGWG domain which is presumed to have a structural function (Shaw, 1992; Suzuki *et al.*, 2003; Bayer *et al.*, 2004; Molina and Kosma, 2015). Formation of acetyl esters of monolignols has also been proposed to be catalysed by members of the BAHD acyltransferase family during phenylpropene production. The only functionally well-characterized acyltransferase involved in phenylpropene synthesis is coniferyl alcohol acetyltransferase (CFAT) from *Petunia*, PhCFAT, a BAHD enzyme which is required for the biosynthesis of isoeugenol, a prominent floral scent component. An RNAi study, determination of the tissue-specific expression pattern, along with biochemical characterization confirmed the role of PhCFAT in formation of coniferyl acetate for isoeugenol synthesis (Dexter *et al.*, 2007). Another acyltransferase was isolated from creosote bush (*Larrea tridentate*), LtCAAT1, using degenerate primers based on conserved sequences among BAHD members. *In vitro* characterization of LtCAAT1 showed its substrate versatility as it could utilize various monolignol substrates including coniferyl alcohol. Heterologous expression of LtCAAT1 in *Escherichia coli* along with *L. tridentata* allylphenol synthase 1 (LtAPS1) resulted in the formation of eugenol (Lu *et al.*, 2017).

In sweet basil, labelling experiments with isolated glandular trichomes have indicated an esterified form of coniferyl alcohol serving as an intermediate for eugenol synthesis. Eugenol synthase (EGS), an NADPH-dependent reductase, has been identified that uses coniferyl acetate and NADPH as substrates to produce eugenol (Koeduka *et al.*, 2006). However, to date, the enzyme responsible for acetylation has not yet been characterized. In this study, we performed high-throughput RNA sequencing (RNA-Seq) of different tissues of sweet basil: namely PGT, leaf minus PGT (leaf–PGT), leaf, and root to generate a quantitative and comparative profile of the gene expression pattern in the PGTs. From the various acyltransferase transcripts identified, we chose two transcripts, namely ObCAAT1 and ObCAAT2, for further characterization. ObCAAT1 was the only alcohol acyltransferase that showed high expression in PGTs, while ObCAAT2 expression in PGTs was very low but it exhibited the highest protein sequence similarity to *Petunia* PhCFAT. We cloned the full-length cDNAs of *ObCAAT1* and *ObCAAT2*, and sequence analysis showed their similarity to the BAHD acyltransferase family of proteins. *In vitro* and *in vivo* characterization of ObCAAT1 and ObCAAT2 demonstrated their ability to produce eugenol from coniferyl alcohol in a coupled reaction with eugenol synthase. Additional biochemical characterization revealed their activity towards other alcohols such as phenylethyl alcohol, cinnamyl alcohol, and benzyl alcohol, indicating substrate versatility. We further functionally characterized the role of *ObCAAT1* in sweet basil PGTs by generating *ObCAAT1*-RNAi lines. Suppression of *ObCAAT1* expression led to a decrease in eugenol content in transgenic lines and accumulation of coniferyl alcohol and its derivatives. Total lignin content in leaves and trichomes of transgenic lines showed no deviation with respect to wild-type sweet basil. This suggests that *ObCAAT1* suppression does not enhance flux into the lignin biosynthesis pathway. Instead, sweet basil chemically modifies the generated accumulated coniferyl alcohol, which is toxic to cells, to form possibly non-toxic derivatives. This indicates that phenylpropene biosynthesis is not coupled to lignification in sweet basil. These results provide insight into the production of eugenol, an economically significant phenylpropene in sweet basil, and increase our understanding of coniferyl alcohol metabolism in plants.

## Materials and methods

### Plant material and transformation

Commercial sweet basil (*O. basilicum*) plants were grown in a greenhouse under natural light conditions. *Agrobacterium*-mediated transformation of sweet basil was performed as previously described (Wang *et al.*, 2016). Briefly, *O. basilicum* seeds were sterilized with 40% Clorox for 3 min, washed with sterile water, and imbibed overnight at 4 °C. The following day, mature embryos were dissected out of the seeds under a dissection microscope. The harvested embryos were pre-cultured in co-cultivation medium (CC) [Murashige and Skoog (MS) salts+myo‐inositol (100 mg l^–1^)+sucrose (30 g l^–1^)+benzyladenine (BA; 0.4 mg1^–l^)+indole-3-butyric acid (IBA; 0.4 mg 1^–l^)+cefotaxime (150 mg 1^–l^)] in the dark dark for 1 d. The pre-cultured embryos were immersed in *Agrobacterium tumefaciens* (EHA105 strain) culture and sonicated for 15 s, four times. Later, the embryos were immersed in fresh *Agrobacterium* solution and vacuum‐infiltrated for 3 min. After infection for 30 min, the embryos were placed in CC medium for 3 d. Subsequently, the embryos were rinsed multiple times with sterile distilled water containing cefotaxime (150 mg1^–l^). The washed embryos were transferred to CC medium for 3–4 weeks in the dark for shoot induction. After 3–4 weeks, green fluorescent protein (GFP)‐positive shoots were selected and transferred to light and later transferred to elongation medium [MS salts+sucrose (30 g 1^–l^)+BA (3 mg1^–l^)+indole-3-acetic acid (IAA; 0.5 mg1^–l^)+cefotaxime (150 mg1^–l^)] and kept for 2–3 weeks. The shoots were hardened which allowed for root formation on basal medium. Plantlets with well‐developed roots were transferred to soil and grown under glasshouse conditions before further analysis. T_1_ and T_2_ transgenic plants were selected using GFP as a visual marker, and T_2_ plants were used for all the experiments. Twenty-day-old *Nicotiana benthamiana* seedling was used for agroinfiltration with *A. tumefaciens*.

### PGT and RNA isolation

Leaves of 3–4 cm were soaked for 1 h in ice-cold imbibition buffer as described previously (Lange *et al.*, 2000). Extraction buffer as described in Lange *et al.* (2000) was added to the soaked leaves (~3 g) in a fresh Falcon tube. For trichome isolation by the glass bead abrasion method, glass beads (Sigma 425–600 μm) were added to the Falcon tube, vortexed for 30 s twice, and passed through a cell strainer (100 μm) to collect the flow through. This was again sieved with a 40 μm cell strainer for collection of PGTs. The accumulated PGTs were washed a few times with isolation buffer, collected using RNase-free water, spun to remove excess water, and frozen in liquid nitrogen. To obtain leaf–PGT, similar stage leaves were brushed with imbibition buffer to remove trichomes and checked under a dissection microscope. Total RNA was extracted from isolated PGT, leaf, leaf–PGT, and roots with the Spectrum™ Plant total RNA kit (Sigma). RNA quality was checked by measuring the OD_260_:OD_280_ ratio. The RNA integrity number (RIN) was determined using an Agilent 2100 bioanalyser.

### Sequencing and assembly

To facilitate RNA sequencing, RNA libraries of PGT, leaf, leaf–PGT, and roots were prepared using the TruSeq RNA Sample Preparation Kits v2; set A (RS-122-2001, Illumina Inc.) according to the manufacturer’s instructions. The Agilent 2200 TapeStation system (Agilent Inc.) was used to evaluate the quality and size of cDNA libraries. The libraries were run on single lanes for 100 cycles (paired-end) on Hiseq™ 2000 (illumine Inc.), independently. Raw reads were analysed for quality check by FastQC (http://www.bioinformatics.babraham.ac.uk/publications.html) that displayed high-quality reads with Q>20. For generating unigenes, *de novo* assembly of the raw reads was completed by the Trinity method (Grabherr *et al.*, 2011). Unigene function annotation was based on sequence similarities to sequences in the public nr database (National Centre for Biotechnology Information) and also the protein sequence databases from *Arabidopsis thaliana*, *Vitis vinifera*, and *Oryza sativa*. The Gene Ontology (GO) terms were retrieved by Trinote from the Gene Ontology database (Grabherr *et al.*, 2011).

### Quantitative real-time PCR (qRT-PCR)

Total RNA (1 µg) isolated from PGT, leaf, leaf–PGT, and roots was reverse transcribed using the iScript™ cDNA Synthesis kit from Bio-Rad (CA, USA). qRT-PCR was performed on an ABI 7900 HT fast real-time system (Life Technologies) using SYBR Green Real-time PCR Master Mixes (Life Technologies). All reactions were performed in triplicate with three biological replicates, including non-template control. For reverse transcription–PCR (RT–PCR), genes were amplified in a T100™ Thermal Cycler (Bio-Rad) by the following program, 94°C for 3 min; 27 cycles of 94°C for 30 s, 60°C for 30 s, 72°C for 20 s; and 72°C for 3 min. The primers used for RNA detection of target genes by qRT-PCR and RT–PCR are listed in Supplementary Table S1 at *JXB* online. The stretches of sequence showing least homology with the other *ObCAAT* genes were chosen for primer design for selective amplification of each *ObCAAT*. The sweet basil elongation factor (*ObEF1α*) gene was used as internal control as it exhibited similar expression in different tissues in our RNA-Seq data. Error bars represent the mean ±SE.

### Gene amplification and plasmid construction

Full-length ORFs encoding *ObCAAT1* and *ObCAAT2* were obtained by carrying out amplification with full-length primers using the Phusion High-Fidelity DNA Polymerase (NEB) kit. Amplified products were cloned into gateway donor vector pENTR™/D- TOPO^®^ (Invitrogen) from Clontech (CA, USA) and the ORFs were sequenced for confirmation. For *ObCAAT1*-RNAi, four primers with restriction enzymes located at their flanking regions were used to amplify the sense and antisense fragments along with an intron in between the two. The purified PCR products were cloned into gateway donor vector pENTR™/D-TOPO^®^ (Invitrogen) and further introduced into the destination vector pK7WG2D via LR recombination. The *ObCAAT1* RNAi gene was driven by the 35S promoter. The destination plasmid construct was transformed into the *A. tumefaciens* strain used for plant transformation. Sequences of primers used in this study are listed in Supplementary Table S1.

### Selection of transgenic lines

GFP selection was used to identify transgenic plants. To confirm gene insertion, DNA was isolated from GFP-positive plants and checked using PCR. DNA-positive lines were then subjected to Southern blot using the digoxigenin (DIG) wash and block buffer set from Roche (IN, USA). The PCR DIG probe synthesis kit from Roche (IN, USA) was used to generate the DNA probe against the *Cauliflower mosaic virus* (CaMV) 35S promoter (Hart and Basu, 2009). Around 20 µg of genomic DNA was digested at 37 °C with *Nde*I for 18 h. Digested product was electrophoresed on a 1% (w/v) agarose gel at 40 V for 5 h. Next, the gel was transported to a nylon membrane and hybridized with the CaMV 35S promoter probe using a DIG DNA labelling and detection kit (Roche). This was followed by antibody binding and chemiluminescent reaction change by CDP star. Lastly, a film was used for exposing the membrane for analysis of the number of T-DNA insertions visualized by the ChemiDoc™ Touch Imaging System (Bio-Rad).

### Extraction of volatiles and GC-MS analysis

Sweet basil plants were grown to obtain the T_1_ and T_2_ generation, and T_2_ plants were used for metabolite analysis. Terpene and phenylpropanoid production in leaves of sweet basil were determined using a GC-MS method. Leaves of 3–4 cm at the fourth node were ground in liquid nitrogen and homogenized using 500 µl of ethyl acetate. A 20 μg aliquot of diethyl sebacate was added as an internal standard for sweet basil. Homogenized samples were incubated for 30 min at room temperature with forceful shaking followed by centrifugation at 13 000 rpm for 10 min. The top organic layer was separated and dehydrated using anhydrous sodium sulfate. The sample extracts collected after extraction were analysed using GC-Qtof 7200 equipped with a HP-5MS fused silica column with helium as carrier gas. The oven was set at a temperature program of 50 °C for 1 min and increased at a rate of 8 °C min^–1^ to 300 °C and held for 5 min. The detector temperature was 280 °C with a mass range from 45 to 450 *m/z*, with an electron energy of 70 eV. Compound identification was done via the NIST library mass spectral database ​using Mass hunter data acquisition software. Quantification of compounds was performed with the standards at different concentrations to establish calibration curves. Three biological replicates were used for GC-MS analysis.

### LC-MS and coniferyl alcohol derivative analysis

For analysis of monolignol derivatives, leaves of 3–4 cm at the fourth node were dried by lyophilization. Dried leaf material was pulverized and 100 mg of ground tissue was subjected to ultrasonication in 2.0 ml of 80% (v/v) methanol for 1 h. Dichloromethane and water in a 1:1 ratio were added to the mixture, vortexed, and centrifuged. The apolar dichloromethane layer was separated from the water phase, evaporated to dryness,

residues were dissolved in methanol, and the methanol extract was analysed by HPLC (van Uden *et al.*, 1991). For each line, three plants were used. Analysis was performed using a micrOTOF-Q™ II LC system (Bruker Daltonics, Bremen, Germany) coupled with a mass spectrometer equipped with a dual electrospray ionization (ESI) source and MS workstation 8.2.1. The UHPLC system included a binary pump, vacuum solvent degasser, autosampler with 108-vial well-plate trays, and a thermostatically controlled column compartment. A ChromolithPerformance RP-18e (2.0×100 mm, Merck) reversed phase column was used and the mobile phase was water with 1.0% formic acid and 10 mM ammonium acetate (A) and methanol (B) with a flow rate of 0.3 ml min^–1^. The linear gradient elution was programmed as –10.0 min, 5% B; 0 min, 5% B; 7 min, 50% B; 10 min, 95% B; 20 min, 95% B; 21 min, 5% B; 25 min, 5% B. The spectra were acquired in the negative ion mode over an *m/z* range from 50 amu to 1200 amu. The MS/MS analyses were carried out by automatic fragmentation and the data were processed through Data Analysis 4.0 software (Bruker, Daltonics). The spectra were also acquired in the positive ion mode over an *m/z* range from 50 amu to 1200 amu. The MS/MS analysis was performed by automatic fragmentation, and the data were processed through Data Analysis 4.0 software (Bruker, Daltonics). For quantification, a coniferyl alcohol calibration curve with a series of standard solutions was used. The results were analysed using the XCMS software. Three biological replicates were used for LC-MS analysis.

### PGT isolation and lignin measurement

Leaves of 3–4 cm in size were brushed with imbibition buffer similar to as described in (Lange *et al.*, 2000) in a 50 ml Falcon tube to harvest trichomes, and checked under a dissection microscope. Total lignin content was measured in isolated trichomes and leaves of *ObCAAT1*-RNAi lines using the thioglycolic acid method (Bonawitz *et al.*, 2014). Briefly, 100 mg of fresh sweet basil leaves and isolated PGTs were ground in liquid nitrogen and extracted in 10 vols of 100% methanol for 2 h at 80 °C. After centrifugation, 10 vols of distilled water were used for washing and samples were incubated for 3 h at 80 °C in a mixture of 750 ml of distilled water, 250 ml of concentrated HCl, and 100 ml of thioglycolic acid. Samples collected after 3 h of centrifugation were washed with 1 ml of distilled water and incubated in 1 ml of 1 M NaOH for 12 h at room temperature with gentle shaking. Samples were subjected to spinning at maximum speed for 10 min, and 200 ml of concentrated HCl was added to the supernatant, vortexed, and incubated at 4 °C for 4 h. Precipitates collected after spinning for 10 min at maximum speed were dissolved in 1 ml of 1 M NaOH, and the absorbance was determined at 280 nm using a spectrophotometer.

### Subcellular localization

The full-length cDNA of *ObCAAT1* and *ObCAAT2* without the stop codon was cloned into the gateway donor vector pENTR™/D-TOPO^®^ (Invitrogen, Germany). The recombinant plasmids were introduced into the destination vector pBA-DC-YFP via LR recombination. pBA-DC-YFP containing the CaMV 35S promoter and the C-terminus in-frame with yellow fluorescent protein (YFP) was used to generate ObCAAT1–YFP and ObCAAT2–YFP. These constructs were then introduced into *A. tumefaciens* strain EHA105 by heat shock. Twenty-day-old *N. benthamiana* seedlings were used for agro-infiltration, and YFP fluorescence was observed by using a confocal microscope after 2 d.

### Expression, purification, and coupled *in vitro* reaction of ObCAAT1, ObCAAT2, and ObEGS1

Full-length ORFs encoding *ObCAAT1*, *ObCAAT2*, and the sweet basil eugenol synthase gene (*ObEGS1*) were cloned into pDEST™20 (Thermo Fischer) to generate the glutathione *S*-transferase (GST)-fused protein. pDESCAAT1, pDESCAAT2, and pDESEGS1 constructs were transformed into *E. coli* BL21 (DE3) by the heat shock method. Expression was induced by adding 1 mM isopropyl-β-d-thiogalactopyranoside (IPTG) followed by incubation at 16 °C for 12 h for ObCAAT1 and ObCAAT2. For ObEGS1, 0.4 mM IPTG was used for induction at 37 °C for 2 h. Recombinant protein was purified using glutathione–Sepharose beads (GE Healthcare) by affinity chromatography according to the manufacturer’s instruction using 20 mM glutathione (GE Healthcare). Coupled reactions involving ObCAAT1 and ObEGS1 and ObCAAT2 and ObEGS1 were determined independently in 100 mM MES KOH buffer (pH 6) using coniferyl alcohol as substrate as previously described by Dexter *et al.* (2007). The reaction mixtures comprised coniferyl alcohol (400 μM), acetyl-CoA (250 μM), NADPH (400 μM), and purified ObCAAT1 or ObCAAT2 and ObEGS1 (5 μg) in autonomous reactions carried out for 60 min at room temperature. A similar *in vitro* coupled reaction was repeated with GST alone in place of ObCAAT as a negative control.

### Transient expression studies of ObCAAT1 and ObCAAT2 in *N. benthamiana* plants

Twenty-day-old *N. benthamiana* seedling were used for agro-infiltration. *Agrobacterium tumefaciens* transformed with plasmids expressing 35S::ObCAAT1, 35S::ObCAAT2, the silencing suppressor 35S::p19, and 35S::ObEGS1 independently were grown overnight, pelleted, and suspended in MMA solution to obtain OD_600_=1. *Nicotiana benthamiana* leaves were infiltrated in batches of two. One batch was co-infiltrated with *A. tumefaciens* suspensions harbouring plasmids expressing 35S::ObEGS1 and the silencing suppressor 35S::p19, together with 35S::ObCAAT1 or 35S::ObCAAT2. The second batch of *N. benthamiana* leaves were infiltrated with the silencing suppressor 35S::p19, together with 35S::ObCAAT1 or 35S::ObCAAT2 independently. The infiltrated plants were incubated for 2 d in a growth chamber with a 16 h photoperiod at 25 °C. Two days post-infiltration, the leaves were re-infiltrated with various alcohols including coniferyl alcohol, hexanol, cinnamyl alcohol, phenylethyl alcohol, and benzyl alcohol (2 mg ml^–1^), and incubated for 1 d in a growth chamber. Further, the infiltrated leaves were ground in liquid nitrogen and homogenized using 2 ml of hexane. Homogenized samples were incubated for 1 h at room temperature with shaking followed by centrifugation at 13 000 rpm for 10 min. The top organic layer was separated, dehydrated using anhydrous sodium sulfate, and concentrated using nitrogen gas.Following concentration, 2 µl of samples were injected for GC-MS and analysed as explained above. Silencing suppressor p19, together with ObCAAT1- or ObCAAT2- and ObEGS1-co-infiltrated leaves, and p19 along with either ObCAAT1- or ObCAAT2-infiltrated leaves with no external alcohol were used as one set of negative controls. Coniferyl alcohol-, cinnamyl alcohol-, hexanol-, phenylethyl alcohol-, and benzyl alcohol-infiltrated leaves without infiltration of ObCAAT1 or ObCAAT2 or ObEGS1 were used as a second set of negative controls.

### Phylogenetic analysis

A total of 124 BAHD sequences comprising both characterized and uncharacterized BAHD sequences (112) belonging to the seven clade-based classification reported in Moglia *et al.* (2016) along with 12 BAHDs (retrieved by the Blastp algorithm) showing close homology with ObCAAT1 and ObCAAT2, including ObCAAT1 and ObCAAT2, were aligned with the ClustalW program (http://www.ebi.ac.uk) using default parameters. A Neighbor–Joining tree was constructed, and bootstrap analysis with 1000 replicates was carried out using MEGA7 and visualized with the FigTree graphical viewer.

### Statistical analysis

Data are indicated as mean ±SE comprising 3–6 biological replicates each performed in triplicate. Statistical significance between transgenic plants and the wild type was analysed using a two-tailed Student’s *t*-test.

### Accession numbers

Sequence data for *ObCAAT1* and *ObCAAT2* have been deposited in GenBank under the accession number MN031888 and MN031889. RNA-Seq data of PGT, leaf–PGT, leaf and root have been submitted to the NCBI SRA under Bioproject number PRJNA54736.

## Results

### Volatile profile of PGTs of *O. basilicum* L. Italian

The sweet basil leaf surface possesses non-glandular multicellular hair-like trichomes and glandular capitate and peltate trichomes. The head of the PGT consists of four secretory cells which produce and secrete the essential oil into the storage cavity (Supplementary Fig. S1A–C). Studies indicate that the composition and concentration of volatiles accumulating in basil (*O. basilicum* L.) PGTs depends on the leaf developmental stage and declines as the leaves mature (Deschamps and Simon, 2010). We performed GC-MS analysis of young (3–4 cm) leaves of sweet basil and found the essential oil to be a mix of phenylpropene and terpenes. Eugenol was the most abundant phenylpropene, followed by monoterpene, eucalyptol and sesquiterpene, and α-bermagotene. In addition to this, monoterpenes such as α-pinene, β-pinene, and linalyl acetate, and sesquiterpenes such as germacrene- D, γ-muurolene, and β-copaene were also detected (Supplementary Fig. S1D). This indicated dynamic metabolic biosynthetic activity of PGTs in leaves at this stage of development. Hence, PGTs were purified from leaves of this stage and RNA was isolated. As a control, the leaves of the same stage were brushed to remove all trichomes and RNA was extracted.

### Sequencing, *de novo* assembly, and annotation of the transcriptome

Four RNA libraries from PGT, leaf–PGT, root, and leaf were prepared and sequenced by Illumina technology. More than 100 million high quality reads of 101 bp were generated (Supplementary Fig. S2). Using the Trinity method, the sequence reads were finally assembled into 102 213 non-redundant unigenes, spanning a total of 101 Mb of sequence with a GC content of 40.89%. All unigenes were longer than 200 bp. The N50 of the final assembled transcripts was 1817 bp. The unigenes were annotated by performing BLASTX search against various protein databases. Among the 102 213 non-redundant unigenes, 36 796 had at least one hit in the BLASTX search with E-value ≤1e-3. Functional classification of GO terms of all unigenes was performed using Trinotate. In order to calculate the expression level for assembled transcripts, we first mapped reads onto them using Bowtie. RSEM (RNA-Seq by Expectation-Maximization) was used to measure the expression level.

### Identification of a BAHD acyltransferase enriched in PGTs

**Fig. 2.**
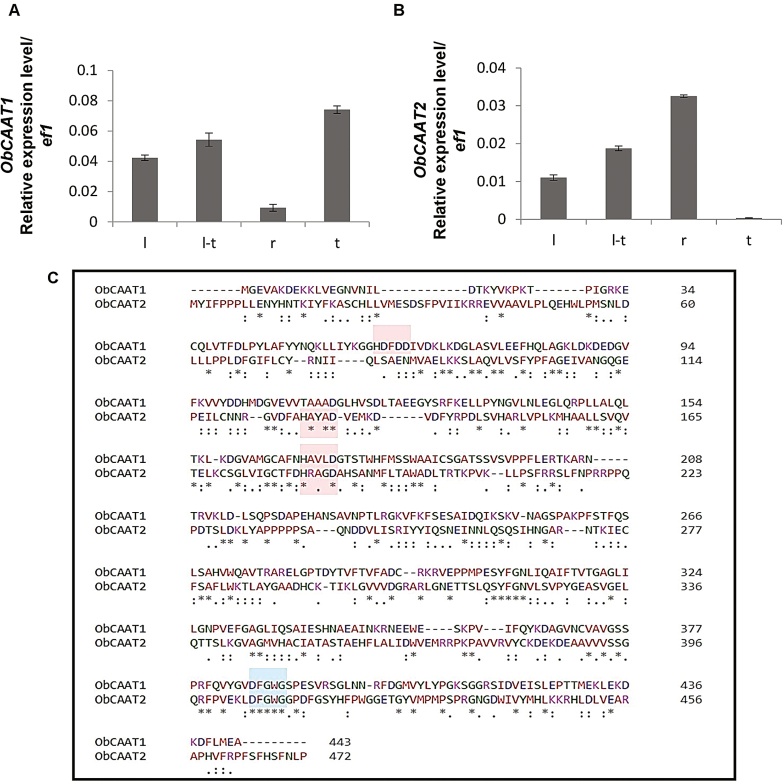
Tissue-specific expression profile and protein sequence alignment of ObCAAT1 and ObCAAT2. qRT-PCR was performed to analyse the expression of (A) *ObCAAT1* and (B) *ObCAAT2* in four different tissues: L, leaf; L-T, leaf stripped of PGTs; R, root; and T, peltate glandular trichome. The housekeeping gene *elongation factor 1* (*ef1*) was used as control. Data are indicated as mean ±SE of three biological replicates each performed in triplicate. (C) HxxxD and DFGWG amino acid motifs, characteristic of BAHD acyltransferase, are present in both ObCAAT1 and ObCAAT2 and are highlighted with boxes.

Analysis of the transcriptome data generated from leaves, leaves–PGT, PGTs, and roots revealed five putative HXXXD-type alcohol acyltransferase transcripts, among which only one, named *ObCAAT1*, showed high expression in PGTs (Supplementary Fig. S3). The alcohol acyltransferase transcript which exhibited the highest homology with the characterized coniferyl alcohol acyltransferase (*PhCFAT*) from Petunia exhibited very low expression in PGTs and was named *ObCAAT2*. The expression pattern of these transcripts as deduced by RNA-Seq was further confirmed by qRT-PCR (Fig. 2A, B).

**Fig. 3.**
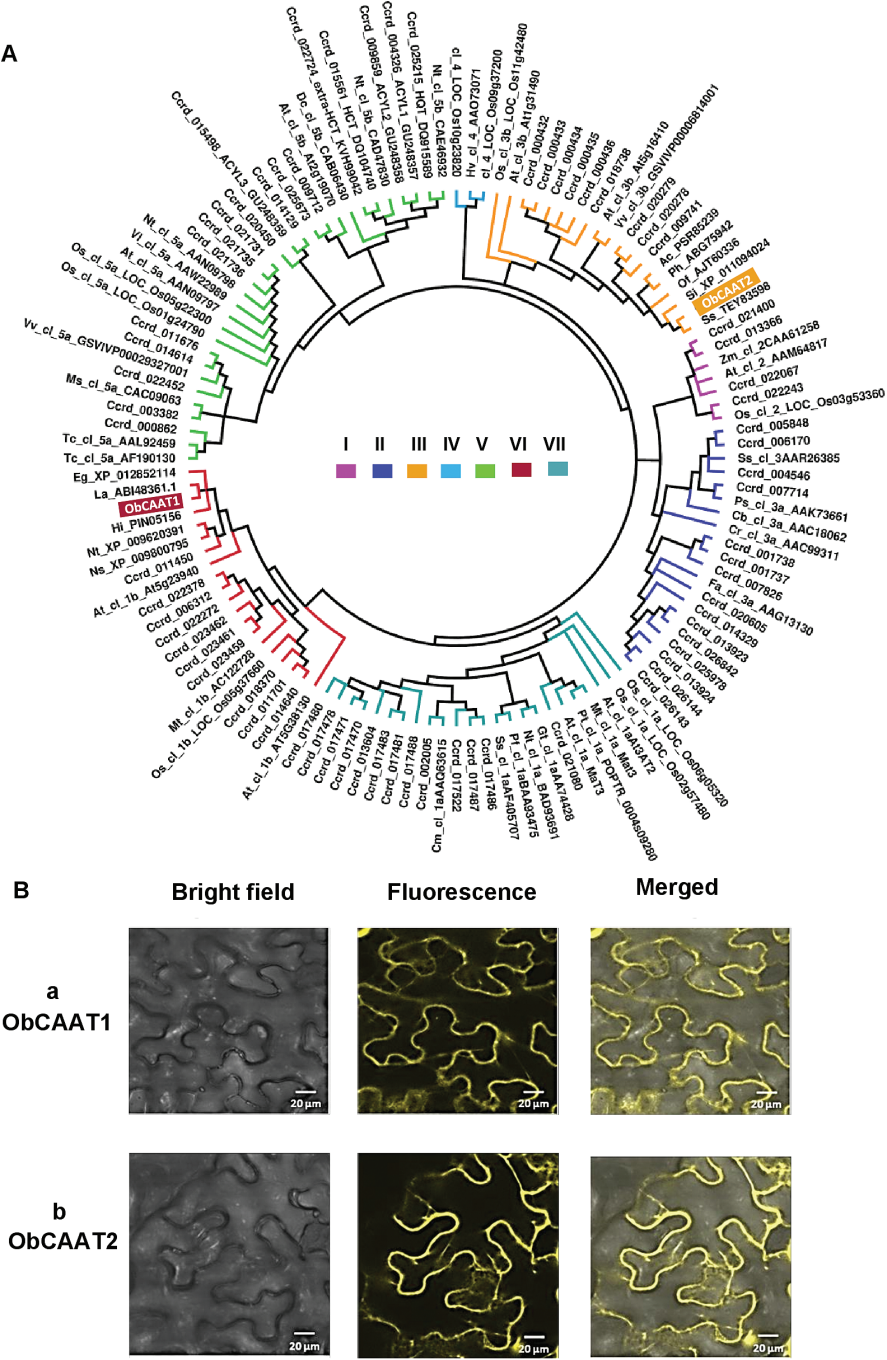
Phylogenetic tree analysis and subcellular localization of ObCAAT1 and ObCAAT2. (A) ObCAAT1 clustered in Clade VI and ObCAAT2 clustered in Clade III. (B) Cytoplasmic localization of (a) ObCAAT1 and (b) ObCAAT2 in *N. benthamiana* leaf cells. Scale bar=20 μm.

The ORFs of *ObCAAT1* and *ObCAAT2* encoded proteins of 444 (48.84 kDa) and 473 (52.03 kDa) amino acids with a calculated isoelectric pH (pI) of 5.43 and 4.95, respectively. Both ObCAAT1 and ObCAAT2 protein sequences possessed the two conserved domains HxxxD and DFGWG, characteristic of all functionally characterized BAHD enzymes (Fig. 2C) (Shaw, 1992; Suzuki *et al.*, 2003; Bayer *et al.*, 2004). Both HxxxD and DFGWG motifs were fully conserved. Two HXXXD motifs were observed in both ObCAAT1 and ObCAAT2. ObCAAT1 showed the highest amino acid sequence similarity of 87.6% with *L. angustifolia* putative alcohol acyltransferase, whereas ObCAAT2 exhibited 73.2% similarity with *Sesamum indicum* shikimate *O*-hydroxycinnamoyl transferase and 61.71% similarity with *P. hybrida* PhCFAT. Phylogenetic analysis was performed with the amino acid sequences of other known BAHD enzymes from different plants (Fig. 3A). ObCAAT1 clustered in Clade VI and ObCAAT2 clustered in Clade III. Interestingly, both Clade III and VI correspond to the group of proteins lacking any functionally defined homologue. Subcellular localization of the proteins was done by transient expression of 35S::ObCAAT1–YFP and 35S::ObCAAT2–YFP fusion proteins in *N. benthamiana* leaves by agroinfiltration, which revealed cytosolic localization of both ObCAAT1 and ObCAAT2 (Fig. 3B). Further, analysis of ObCAAT1 and ObCAAT2 sequences did not reveal any transit/signal peptide, thus supporting the absence of organelle localization or secretion as observed in the majority of BAHD members characterized to date (D’Auria, 2006).

### *In vitro* and *in vivo* coupled reaction of ObCAAT1 and ObCAAT2 with ObEGS1 converts coniferyl alcohol to eugenol

**Fig. 4.**
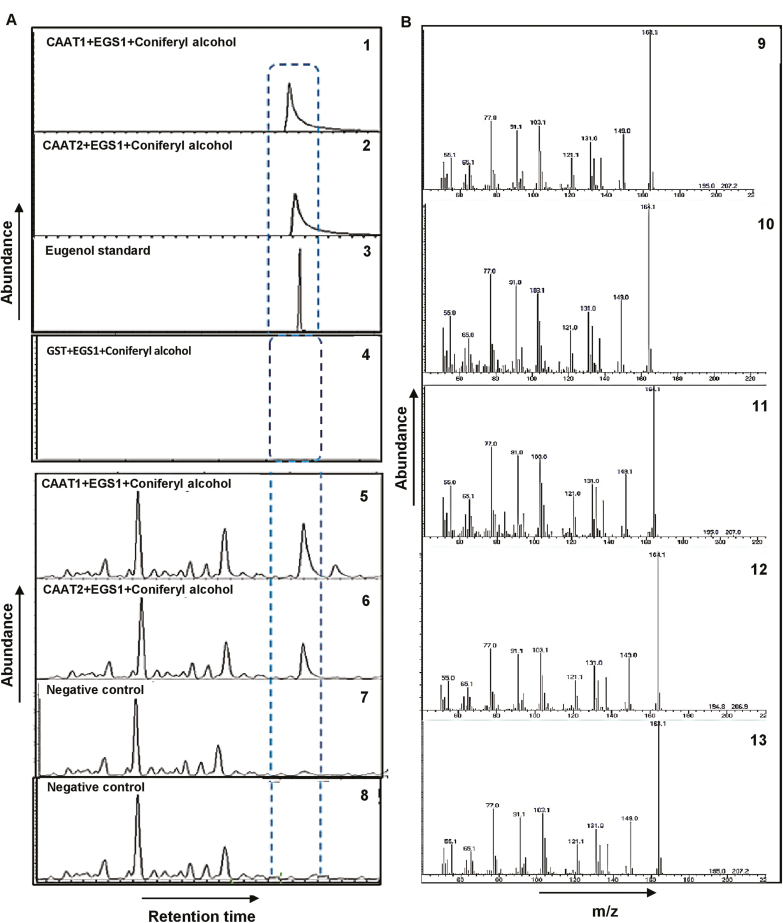
(A) *In vitro* functional characterization of ObCAAT1 and ObCAAT2. (A) GC-MS analysis of products formed *in vitro* by purified ObCAAT1, ObCAAT2, and ObEGS1 in a coupled reaction. (1) Eugenol was produced by purified ObCAAT1 and ObEGS1 using coniferyl alcohol as the substrate. (2) Eugenol was produced by purified ObCAAT2 and ObEGS1 using coniferyl alcohol as the substrate. (3) GC-MS profile of eugenol standard. (4) Eugenol was not produced by purified GST and ObEGS1 using coniferyl alcohol as the substrate. (B) GC-MS analysis of products formed by ObCAAT1 and ObEGS1 and ObCAAT2 and ObEGS1 *in planta* in *N. benthamiana* leaves. (5) Eugenol was produced by ObCAAT1 and ObEGS1 using coniferyl alcohol as the substrate. (6) Eugenol was produced by ObCAAT2 and ObEGS1 using coniferyl alcohol as the substrate. (7) Eugenol production was absent in ObCAAT1- or ObCAAT2- and ObEGS1-infiltrated leaves with no external coniferyl alcohol. (8) Eugenol production was absent in coniferyl alcohol-infiltrated leaves without infiltration of ObCAAT1 or ObCAAT2 with ObEGS1. (C) Mass spectrogram of (9) eugenol produced by purified ObCAAT1 and ObEGS1 using coniferyl alcohol as the substrate, (10) eugenol produced by purified ObCAAT2 and ObEGS1 using coniferyl alcohol as the substrate, (11) eugenol produced by ObCAAT1 and ObEGS1 using coniferyl alcohol in *N. benthamiana*, (12) eugenol produced by ObCAAT2 and ObEGS1 using coniferyl alcohol in *N. benthamiana*, and (13) eugenol standard.

ObEGS1 has been characterized to use coniferyl acetate as a substrate to form eugenol. To ascertain the role of putative ObCAAT1 and ObCAAT2 towards the formation of coniferyl acetate and finally eugenol, we performed a coupled *in vitro* enzyme characterization assay. ORFs of *ObCAAT1*, *ObCAAT2*, and *ObEGS1* were expressed in *E. coli* BL21 cells. The recombinant GST-tagged fusion protein was purified by affinity chromatography using glutathione–Sepharose beads. The purified fusion proteins of ObCAAT1, ObCAAT2, and ObEGS1 were observed at ~74, 78, and 60 kDa on SDS–PAGE, which coincided with the calculated molecular mass of these three proteins with the addition of the 26 kDa GST tag. The purified proteins were used in an *in vitro* coupled reaction along with coniferyl alcohol, acetyl-CoA, and NADPH. Both ObCAAT1 and ObCAAT2 in combination with ObEGS1 produced eugenol. As a control, purified GST was used in an *in vitro* coupled reaction along with ObEGS1, coniferyl alcohol, acetyl-CoA, and NADPH. Eugenol was not produced in this coupled reaction, thus negating the possibility of acyltransferase reactions being catalysed by the GST tag in recombinant protein *in vitro* assays (Fig. 4A).

To confirm the enzyme activity of ObCAAT1 and ObCAAT2 *in planta*, *N. benthamiana* plants were used. *Agobacterium tumefaciens* containing plasmids expressing *ObCAAT1* (*35S::ObCAAT1*) or *ObCAAT2* (*35S::ObCAAT2*) with *ObEGS1* (*35S::ObEGS1*) were infiltrated into *N. benthamiana* leaves. After 2 d, infiltrated leaves were analysed by GC-MS and no eugenol production was observed in infiltrated *N. benthamiana* leaves. When the plants were infiltrated with the substrate coniferyl alcohol and analysed by GC-MS, eugenol production was observed, indicating the lack of substrate availability in tobacco leaves (Fig. 4B, C).

### ObCAAT1 and ObCAAT2 display substrate versatility by acetylating other alcohols

To determine if both ObCAAT1 and ObCAAT2 are able to act on other alcohol substrates apart from coniferyl alcohol, they were evaluated for their ability to acetylate alcohols such as hexanol, phenylethyl alcohol, cinnamyl alcohol, and benzyl alcohol *in planta*. For this, *N. benthamiana* leaves were infiltrated with *A. tumefaciens* cultures expressing *ObCAAT1* (*35S::ObCAAT1*) or *ObCAAT2* (*35S::ObCAAT2*). After 2 d, the same leaves were infiltrated with different alcohol substrates such as hexanol, phenylethyl alcohol, cinnamyl alcohol, and benzyl alcohol individually. After 24 h of substrate infiltration, leaves were analysed by GC-MS to check for the production of acetates from the respective alcohols. Infiltration of leaves with only the alcohol substrates did not result in the formation of any acetates in the absence of *ObCAAT1* (*35S::ObCAAT1*) or *ObCAAT2* (*35S::ObCAAT2*), and vice versa. Interestingly, ObCAAT1 and ObCAAT2 displayed common and distinct alcohol substrate utilization. Both ObCAAT1 and ObCAAT2 acetylated benzyl alcohol to benzyl acetate and did not act on hexanol. ObCAAT1- and phenylethyl alcohol-infiltrated leaves produced phenylethyl acetate, in contrast to ObCAAT2 that showed no acetylation of phenylethyl alcohol. Similarly, ObCAAT2- and cinnamyl alcohol-infiltrated *N. benthamiana* leaves produced cinnamyl acetate whereas ObCAAT1 did not (Supplementary Fig. S4A–D).

### *ObCAAT1* suppression leads to decreased eugenol synthesis and accumulation of coniferyl alcohol and its derivatives in sweet basil

**Fig. 5.**
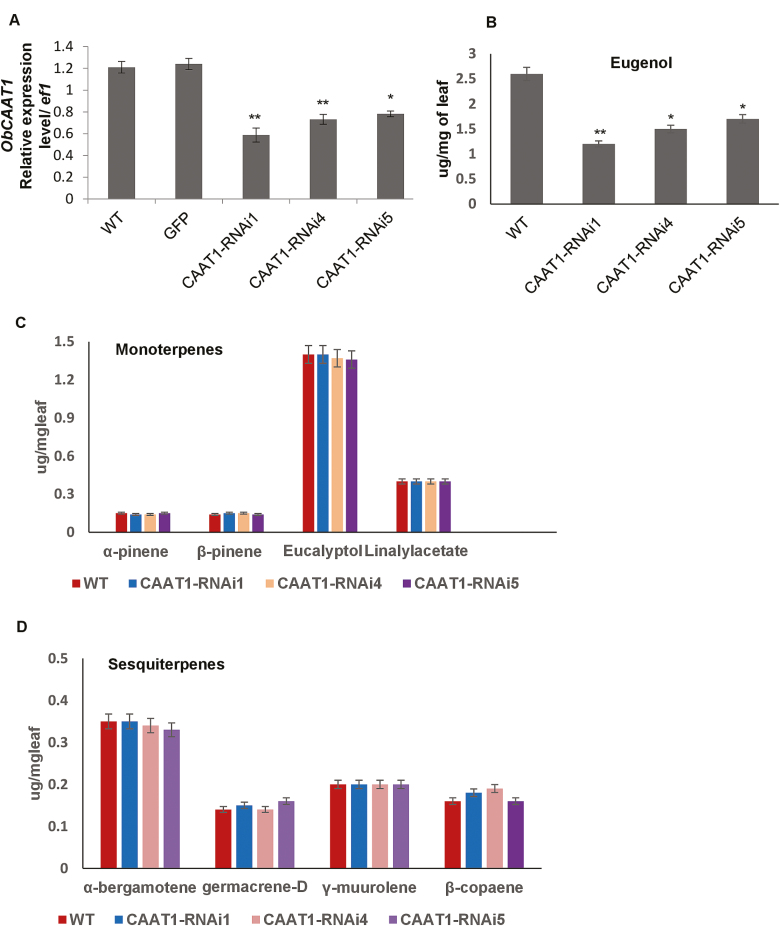
Transcript and metabolite analysis of RNAi transgenic lines. (A) Decreased transcripts level of *ObCAAT1* in transgenic sweet basil *ObCAAT1-*RNAi lines when compared with the wild type (WT) and GFP-overexpressing lines. (B) Reduced levels of eugenol in *ObCAAT1-*RNAi lines when compared with the WT. (C and D) Unaltered levels of monoterpenes and sesquiterpenes in *ObCAAT1-*RNAi lines when compared with the WT and GFP-overexpressing line. Data are indicated as mean ±SE. *P<0.05; **P<0.01; ***P<0.001.

To further characterize the function of *ObCAAT1* in sweet basil, we generated *ObCAAT1-*RNAi lines. Many transgenic lines were generated; out of which three independent transformed lines confirmed by Southern blot were selected for further characterization (*ObCAAT1*-RNAi lines 1, 4, and 5) (Supplementary Fig. S5A). *ObCAAT1* transcript analysis by qRT-PCR revealed a considerable reduction in all transgenic lines compared with controls; wild type and 35S-GFP plants (Fig. 5A). Overall, phenotypically, RNAi transgenic plants looked similar to wild-type plants including the PGTs (Supplementary Fig. S5B). GC-MS analysis was performed to assess qualitative or quantitative changes in the volatile profile of *ObCAAT1*-RNAi lines. GC-MS analysis revealed a substantial decrease in eugenol production in all the three transgenic lines which correlated with the *ObCAAT1* transcript reduction levels. Total eugenol production in *ObCAAT1* transgenic lines was ~45–65% less in comparison with the wild type (Fig. 5B). No difference was observed in levels of monoterpenes such as eucalyptol, linayl acetate, α-pinene, and β-pinene, and sesquiterpene such as α-bergamotene, germacrene D, γ-murrolene, and β-copaene in all the three *ObCAAT1*-RNAi lines (Fig. 5C, D).

**Fig. 6.**
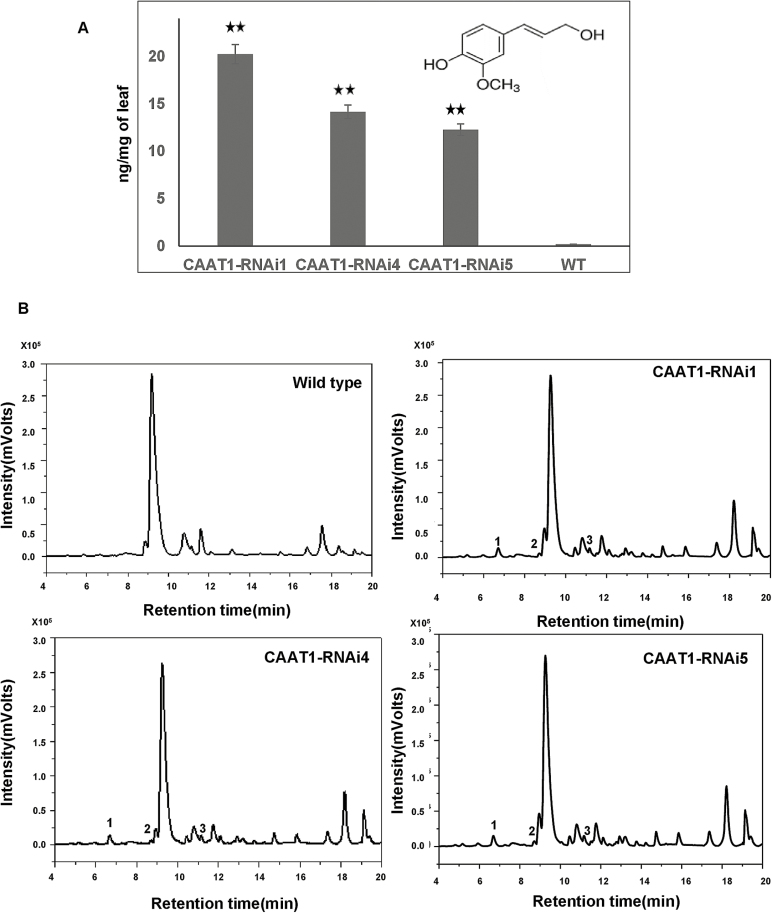
Identification of coniferyl alcohol and its derivatives in *ObCAAT1* transgenic lines. (A) Concentration profile of coniferyl alcohol by LC-Q-TOF MS in the three *ObCAAT1*-RNAi lines. (B) LC-Q-TOF chromatograms depicting coniferyl alcohol and its derivatives in all three *ObCAAT1*-RNAi lines and its absence in wild-type plants. (1) Coniferyl alcohol, (2) 4-hydroxy-3-methoxycinnamate, and (3) dihydroxy methoxy flavone in ObCAAT1 transgenic lines. Data represent the mean ±SE for three biological replicates. (*P≤0.05; **P≤0.01; ***P≤0.001).

Monolignol coniferyl alcohol is a shared intermediate between eugenol and lignin biosynthesis. To confirm if suppression of the eugenol pathway results in channelling of coniferyl alcohol into the lignin pathway, total lignin analysis of transgenic lines was performed. The amount of total lignin was estimated in leaves and isolated PGTs of *ObCAAT1*-RNAi lines by thioglycolic acid and found to be similar to that of wild-type plants (Supplementary Fig. S6). This indicates that knock down of *ObCAAT1* in sweet basil has no effect on the lignin biosynthesis pathway. Monolignols such as coniferyl alcohol are known to be toxic to cells in high concentration and are metabolized to non-toxic derivatives. To detect the presence of such derivatives, detailed GC-MS and LC-MS analyses of the transgenic lines were performed. Both GC-MS (Supplementary Fig. S7) and LC-MS analysis (Fig. 6A, B) revealed the presence of coniferyl alcohol in the transgenic lines not observed in wild-type plants. Based on the relative retention time and total ion mass spectral comparison with the coniferyl alcohol standard, the presence of coniferyl alcohol was identified in the transgenic lines. The mass spectrum of coniferyl alcohol showed a precursor ion at *m/z* 179 (100%) which was compared with the coniferyl alcohol standard for identification. Previous studies on lignin biosynthesis in angiosperms (Li *et al.*, 2001) and in *Arum italicum* tubers (Della Greca *et al.*, 1993), and a kinetic study of coniferyl alcohol radical binding (Halls *et al.*, 2004) have similarly identified coniferyl alcohol. The amount of coniferyl alcohol in the three *ObCAAT1*-RNAi lines correlated with the reduction in eugenol concentration observed in them. *ObCAAT1*-RNAi line 1 which had the maximum reduction in eugenol synthesis showed the highest concentration of coniferyl alcohol of 20.1 ng mg^–1^, with lines 4 and 5 exhibiting 14.1 ng mg^–1^ and 11.4 ng mg^–1^ DW, respectively. Apart from coniferyl alcohol, LC-MS studies (positive and negative mode) of wild-type and *ObCAAT1*-RNAi lines revealed the presence of putative coniferyl alcohol derivatives such as 4-hydroxy-3-methoxycinnamate (0.4–0.5 ng mg^–1^), dihydroxy methoxy flavone (0.1–0.3 ng mg^–1^), and 1,28-octacosane diol ferulate (0.2–0.3 ng mg^–1^) in all the three transgenic lines (Fig. 6C; Table 1). These metabolites possessed the hydroxy methoxy groups in their structure similar to coniferyl alcohol, indicating the parent connectivity with it. Further, the putative coniferyl alcohol derivatives were identified with the respective precursor ion, mass, and molecular formula (Fabre *et al.*, 2001; Abu-Reidah *et al.*, 2015; Masike *et al.*, 2017). The above results suggest that the suppression of *ObCAAT1* results in accumulation of coniferyl alcohol which possibly is derivatized to prevent toxicity.

**Table 1.** LC-quadrupole time of flight-MS (both positive and negative mode) analysis of coniferyl alcohol derivatives in *ObCAAT1*-RNAi transgenic lines

Transgenic lines	Putative identity	Retention time (min)	Molecular formula	*m/z*	MS/MS fragment
**CAAT1_RNAi1**	*Coniferyl alcohol*	6.56	C10H11O3	179.15	179, 135
	*4-Hydroxy-3-methoxycinnamate*	8.72	C10H9O4	193.05	193,112
	*1,28-Octacosane diol ferulate*	18.82	C48H74O8	778.53	778, 324
	*Dihydroxy methoxyflavone*	11.76	C16H11O5	283.26	283, 112
**CAAT1_RNAi4**	*Coniferyl alcohol*	6.56	C10H11O3	179.15	179, 135
	*4-Hydroxy-3-methoxycinnamate*	8.72	C10H9O4	193.05	193,112
	*Dihydroxy methoxyflavone*	11.77	C16H11O5	283.26	283, 112
**CAAT1_RNAi5**	*Coniferyl alcohol*	6.57	C10H11O3	179.15	179, 135
	*4-Hydroxy-3-methoxycinnamate*	8.72	C10H9O4	193.05	193,112
	*Dihydroxy methoxyflavone*	11.77	C16H11O5	283.26	283, 112

## Discussion

Phenylpropenes contribute to the flavour and aroma of many aromatic spices and herbs, and are commercially valuable as they are widely used in flavours, fragrance, and pharmaceutical industries. Sweet basil (*O. basilicum*) belonging to the Lamiaceae family produces essential oils rich in phenylpropenes such as eugenol, chavicol, and their methylated derivatives. These phenylpropenes are produced and stored in specialized organs known as PGTs. Genetic pathways leading to the formation of these valuable compounds have not been completely elucidated. In sweet basil, the enzymes catalysing the formation of eugenol from coniferyl acetate and its methylated derivatives have been characterized (Gang *et al.*, 2002). Eugenol synthase, a member of the PIP family of NADPH-dependent reductases, catalyses formation of eugenol only from the ester form of monolignol coniferyl alcohol (Kahn *et al.*, 2000; St-Pierre and De Luca, 2000; Koeduka *et al.*, 2006). However, the enzyme that catalyses the acetylation of coniferyl alcohol to produce coniferyl acetate in PGTs has not been characterized. The BAHD acyltransferase family constitute a large group of acyl-CoA-utilizing enzymes that are involved in the modification of several plant secondary metabolites (St-Pierre and De Luca, 2000). Genome and expression analysis of various plant species have revealed the presence of large numbers of BAHD genes performing diverse functions in different tissues within a single species (D’Auria *et al.*, 2002; Hoffmann *et al.*, 2003; D’Auria and Gershenzon, 2005). Analysis of sweet basil transcriptome data from various tissues revealed the presence of many BAHD acyltransferase members having the BAHD family-specific HxxxD motif and DFGWG motif (Ma *et al.*, 2005). However, *ObCAAT1* was the only BAHD family member which had high expression in PGTs, while *ObCAAT2*, which showed highest similarity to *PhCFAT* from Petunia, displayed enriched expression in roots.

The BAHD family has been organized into seven major phylogenetic clades, with members showing both clade-specific and clade-independent biochemical activities (Tuominen *et al.*, 2011; Moglia *et al.*, 2016). Clade I correspond to the group of enzymes involved in extension of the epicuticular wax chain, important for defence and prevention of water loss. Clade II contains acyltransferases that produce volatile esters from a range of alcohol substrates. Proteins lacking any functionally defined homologue are grouped under Clade III and VI. Clade IV proteins show involvement in biosynthesis of anti-fungal hydroxycinnamoyl agmatine derivatives. The enzyme for paclitaxel biosynthesis along with another subset of acyltransferases using hydroxycinnamoyl/benzoyl-CoA as acyl donor are clubbed together in Clade V. Enzymes clustered in Clade VII are involved in modification of phenolic glycosides (Tuominen *et al.*, 2011; Moglia *et al.*, 2016). ObCAAT1 clustered in Clade VI and ObCAAT2 clustered in Clade III, making functional predictions based on sequence difficult.

Coupled *in vitro* and *in planta* (*N. benthamian*) enzymatic characterization of purified ObCAAT1 and ObCAAT2 with EGS using coniferyl alcohol, acetyl-CoA, and NADPH resulted in the formation of eugenol. This indicates the ability of both ObCAAT1 and ObCAAT2 to acetylate coniferyl alcohol to coniferyl acetate which is the first committed step towards eugenol production. The BAHD family includes many enzymes that accept a range of alcohol substrates (Dudareva *et al.*, 1998). *In planta* characterization of ObCAAT1 and ObCAAT2 in *N. benthamiana* leaves with different alcohol substrates showed that they are versatile in substrate specificity. Studies show that BAHD enzymes are capable of acting on a wide range of alcohol substrates *in vitro* to form a variety of metabolites, but the actual metabolites formed in plants by a BAHD enzyme is determined by its specific expression pattern, the availability of substrates, and catalytic efficiency (Peng *et al.*, 2016). Under, *in vitro* conditions and infiltration studies in *Nicotiana*, where the substrate was provided artificially, both ObCAAT1 and ObCAAT2 were able to produce eugenol from coniferyl alcohol, indicating functional redundancy. However, in real native conditions inside sweet basil plants, due to their differential expression pattern, they probably catalyse biologically distinct reactions. ObCAAT1 displayed the highest expression in PGTs, while ObCAAT2 had negligible expression in PGTs. PGTs are the main site of eugenol biosynthesis in leaves, hence PGT-enriched expression of ObCAAT1 indicates its dominant role in the conversion of coniferyl alcohol to coniferyl acetate during eugenol production. This is corroborated by the significant decrease in eugenol formation observed in *ObCAAT1*-RNAi lines. ObCAAT2 showed the highest expression in roots while OBCAAT1 had the lowest expression, suggesting that ObCAAT2 plays a dominant role in roots acting on alcohol substrates that are more abundant in roots for formation of root-specific esters. Additionally, both ObCAAT1 and ObCAAT2 also showed expression in leaf–PGT tissue, pointing towards their activity in these cells. Therefore, in sweet basil, ObCAAT1 and OBCAAT2 might act on similar or different alcohol substrates depending on their tissue-specific expression pattern

*ObCAAT1*-RNAi transgenic lines showed a 45–65% reduction in eugenol biosynthesis. The reduced eugenol production can be attributed to reduced production of the substrate coniferyl acetate available for ObEGS1, thus affirming the role of OBCAAT1 in the conversion of coniferyl alcohol to coniferyl acetate during eugenol production in PGTs. Petunia flowers emitting phenylpropene isoeugenol also displayed a similar inhibition of isoeugenol biosynthesis in *PhCFAT-*RNAi lines (Dexter *et al.*, 2007). Monolignols such as coniferyl alcohol are synthesized in the cytoplasm and, like most BAHD enzymes, ObCAAT1 also showed cytosolic localization and contained no obvious signal peptide sequences. In phenylpropene-producing tissues, coniferyl alcohol is a shared substrate between phenylpropene and lignin biosynthesis (Schilmiller *et al.*, 2008; Muhlemann *et al.*, 2014). Lignin is mainly formed by the polymerization of three types of monoliognols (coniferyl alcohol, G unit; sinapyl alcohol, S unit; and *p*-coumaryl alcohol, H unit). In angiosperms, lignin is normally dominated by syringyl and guaiacyl units (Weng and Chapple, 2010). Analysis of the total lignin content of isolated trichomes and leaves in *ObCAAT1*-RNAi lines did not show any change when compared with the wild type. This suggests that upon suppression of the phenylpropene pathway, the increased levels of the common precursor coniferyl alcohol do not get channelled towards lignin biosynthesis.

Monolignols such as coniferyl alcohol are proposed to be toxic and unstable, because of which they seldom accumulate in large amounts in plant cells (Whetten and Sederoff, 1995; Alejandro *et al.*, 2012; Väisänen *et al.*, 2015; Le Roy *et al.*, 2016). To reduce toxicity, monolignols such as coniferyl alcohol are known to be glycosylated and stored in vacuoles. The glucoside of coniferyl alcohol is known as coniferin and is presumed to be a stored reserve of coniferyl alcohol for future lignification, and also has been reported to be involved in protection against biotic stress such as fungal attack in Arabidopsis (König *et al.*, 2014). No coniferin was observed in *ObCAAT1*-RNAi lines by LC-MS analysis. Apart from lignin and eugenol production, coniferyl alcohol can also serve as a precursor for lignan synthesis. Lignans are metabolites formed by the coupling of two coniferyl alcohol monomers. Pinoresinol, lariciresinol, and secoisolariciresinol are a few common lignans derived from coniferyl alcohol (Suzuki and Umezawa, 2007). These compounds were also not observed in *ObCAAT1*-RNAi lines. In the case of Petunia *PhCFAT* suppression, accumulation of coniferyl aldehyde and its derivative homovanillic acid was observed in the RNAi lines. Comparative LC-MS analysis of the wild type and *ObCAAT1*-RNAi lines showed the presence of coniferyl alcohol and its putative derivatives such as hydroxy-3-methoxycinnamate, 1,28-octacosane diol ferulate, and dihydroxy methoxy flavones. These metabolites feature similar hydroxy methoxy groups in their structure which is present in coniferyl alcohol indicating their derivation from it. The chemical structure of these derivatives indicates that coniferyl alcohol probably underwent a hydroxylation and methylation reaction to form these derivatives. Studies have shown the existence of a pathway in plants involving hydroxylation and subsequent *O*-methylation of coniferyl alcohol and coniferyl aldehyde to generate new metabolites (Chen *et al.*, 1999; Humphreys *et al.*, 1999). Hence, depending upon the enzymatic landscape present in a cell, a single metabolite such as coniferyl alcohol can be metabolized to diverse compounds in different plants (Gao *et al.*, 1998) The physiological effect of these compounds produced in sweet basil remains to be studied.

In conclusion, *ObCAAT1* is the first BAHD acyltransferase gene identified from sweet basil that is involved in eugenol synthesis. This provides new prospective targets for pathway engineering to enhance production of phenylpropenes. This study shows that in response to the limited metabolism of coniferyl alcohol to eugenol upon *ObCAAT1* suppression, coniferyl alcohol is converted to various derivatives to prevent its potential toxic effect.

## Supplementary data

Supplementary data are available at *JXB* online.

Fig. S1. Trichomes on sweet basil leaf surface and composition of sweet basil essential oil.

Fig. S2. Quality of reads and statistics of sequencing.

Fig. S3. Heat map analysis representing differential expression pattern of five BAHD acyltransferases along various tissues.

Fig. S4. *In planta* functional characterization of ObCAAT1 and ObCAAT2.

Fig. S5. Southern blot and phenotypic analysis of *ObCAAT1*-RNAi sweet basil lines.

Fig. S6. Total lignin quantification of *ObCAAT*-RNAi sweet basil lines by the thioglycolic acid method.

Fig. S7. MS spectrum with molecular ion peak at *m/z* 179 for coniferyl alcohol.

Table S1. List of primers used in the present study.

eraa142_suppl_Supplementary_Material

## Abbreviations

BAHD: benzylalcohol *O*-acetyl transferase (BEAT), anthocyanin *O*-hydroxycinnamoyl transferase (AHCT), anthranilate *N*-hydroxycinnamoyl/benzoyl transferase (HCBT), diacetyl vindoline 4-*O*-acetyltransferase (DAT)
ObCAAT: *Ocimum basilicum* coniferyl alcohol acyltransferase
PGT: peltate glandular trichome
RNA-Seq: RNA sequencing
VOC: volatile organic compound
