# Supplementary material for: Characterization of a sweet basil acyltransferase involved in eugenol biosynthesis
Source: J Exp Bot. 2020 Mar 21;71(12):3638–52. doi: 10.1093/jxb/eraa142 (PMC7307857; doi:10.1093/jxb/eraa142)
Supplement: eraa142_suppl_Supplementary_Material [file eraa142_suppl_supplementary_material.pdf]

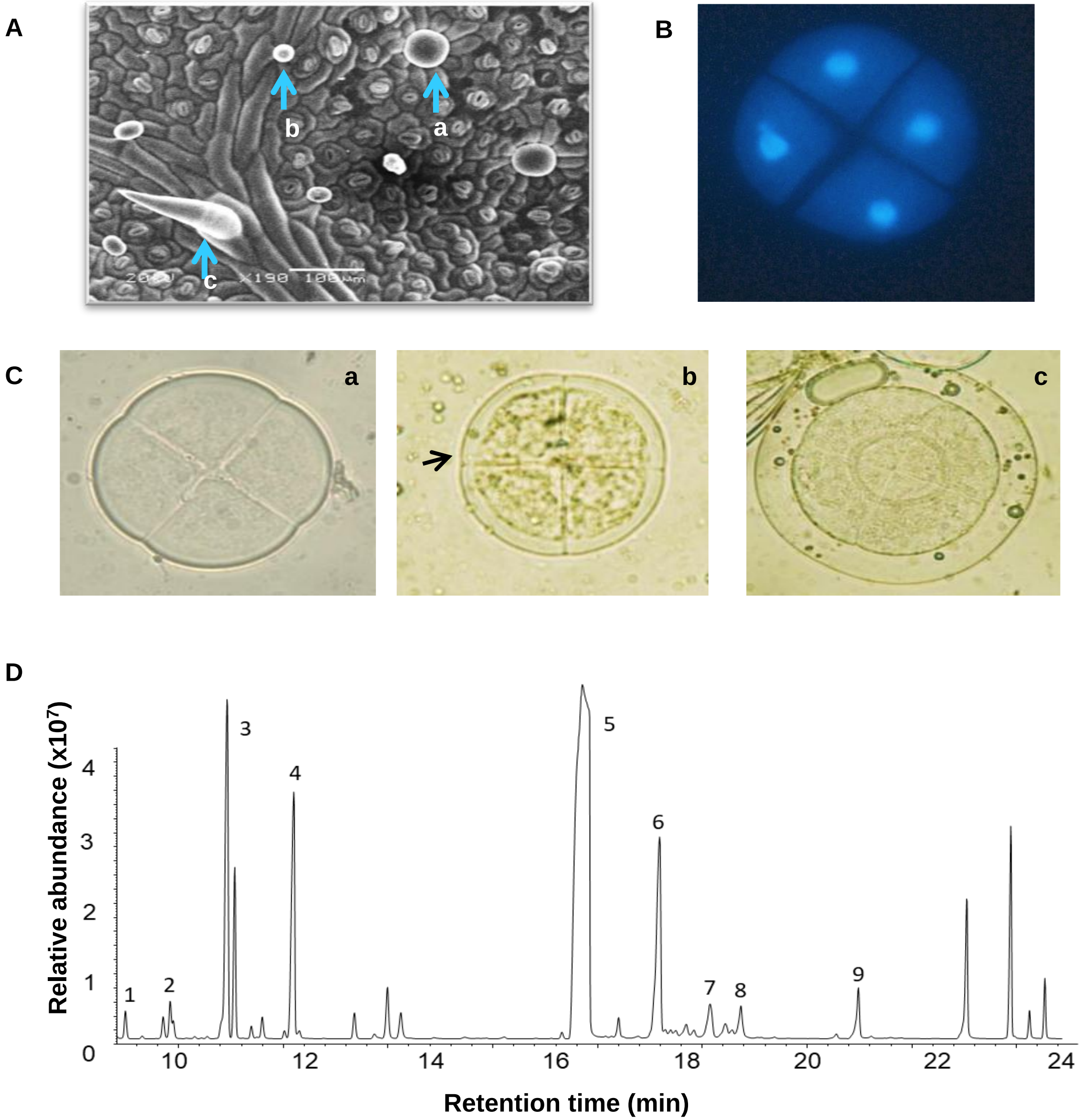

Supplementary Figure 1. Trichomes on sweet basil leaf surface and composition of sweet basil essential oil. (A) Scanning electron microscope image of sweet basil leaf showing three types of trichomes, a, Non glandular hairy trichome; b, Peltate glandular trichome (PGT); c, Capitate glandular trichome. (B) Isolated PGT of sweet basil stained with DAPI. (C) Process of secretion by PGT, a, presecretory stage; b, formation of storage cavity; c, secretion into the storage cavity. (D) GC-MS analysis of sweet basil leaf showing the presence of monoterpenes, 1, α-pinene, 2, β-pinene, 3, Eucalyptol, 4,linalyl acetate, phenylpropene 5, Eugenol, and sesquiterpene, 6, α-bermagotene, 7, germacrene-D, 8, ɣ-muurolene, 9, β-copaene.

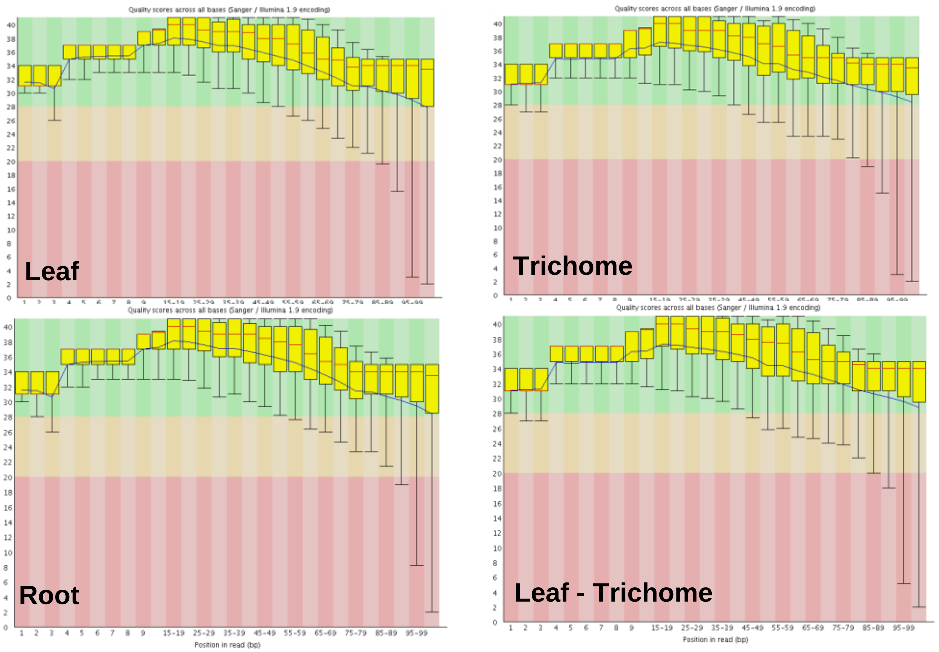

Overview of quality of deep sequencing

Sample	Total Bases	Read Count	N (%)	GC (%)	Q20 (%)	Q30 (%)
**Leaf**	**18,449,677,272**	**182,670,072**	**0.007**	**48.3**	**93.99**	**85.84**
**Root**	**19,013,829,942**	**188,255,742**	**0.007**	**46.46**	**94.38**	**86.62**
**Trichome**	**17,198,322,218**	**170,280,418**	**0.030**	**44.18**	**96.11**	**89.85**
**Leaf-Trichome**	**20,491,806,170**	**202,889,170**	**0.029**	**48.17**	**95.18**	**87.96**

Statistics of deep sequencing

Supplementary Figure 2. Quality of reads and statistics of sequencing

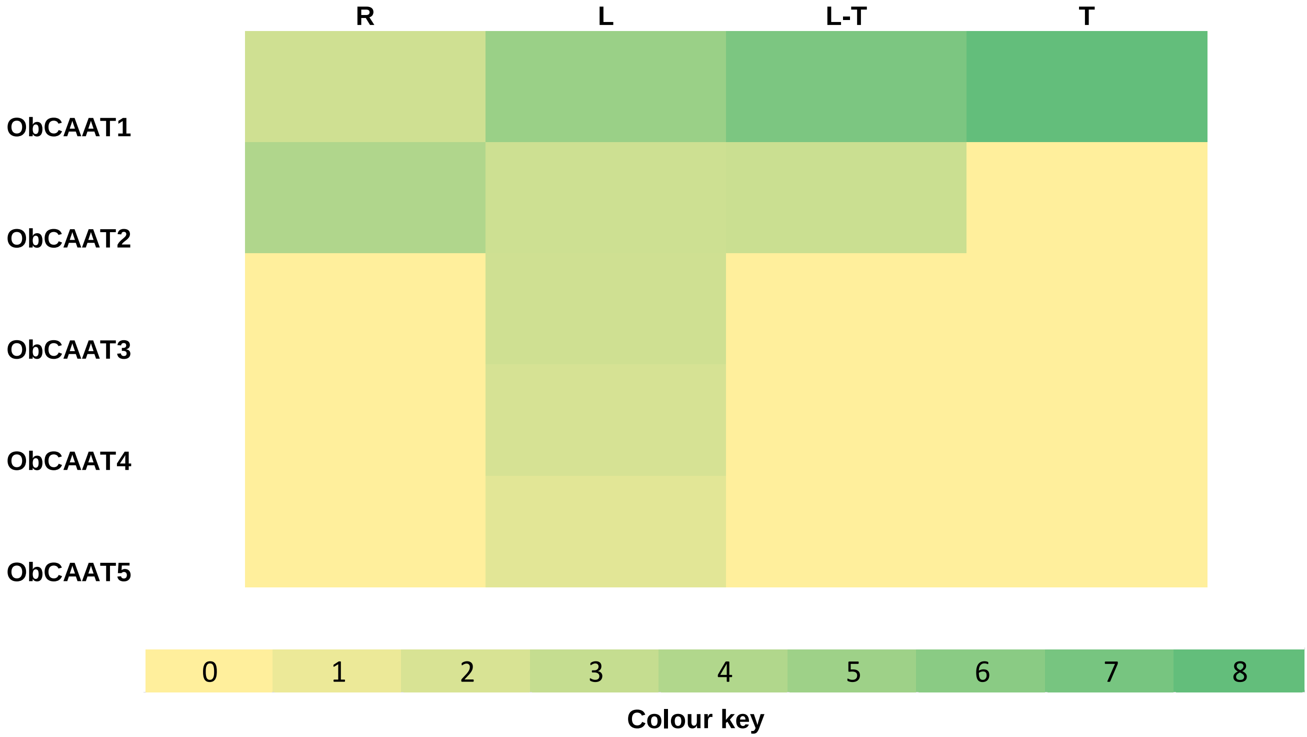

Supplementary Figure 3. Heat map representing differential expression pattern of five BAHD acyltransferases along various tissues [root (R), leaf (L), leaf stripped of PGTs (L-T) and PGTs (T)]

A

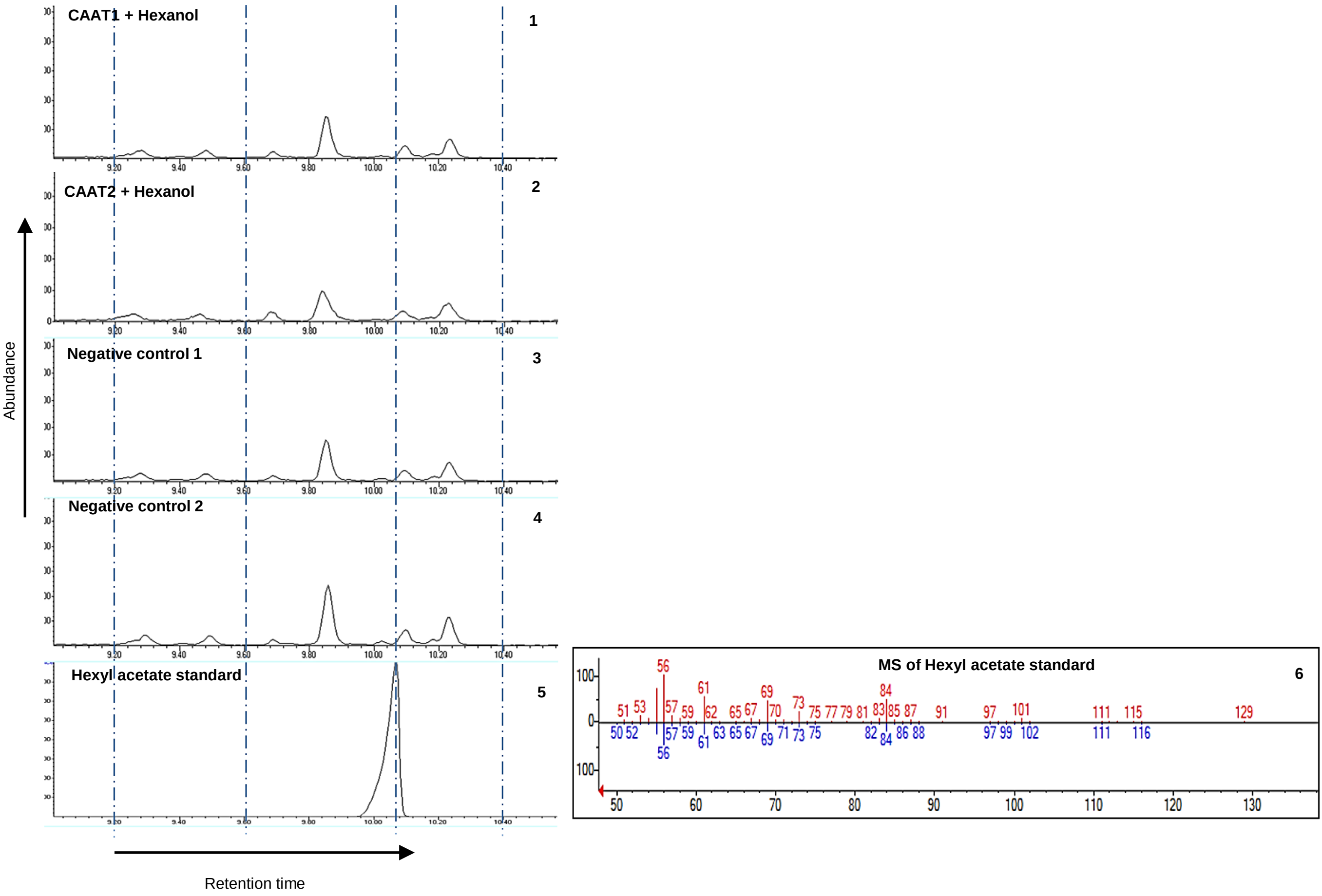
CAAT1 + Hexanol
1
CAAT2 + Hexanol
2
Negative control 1
3
Negative control 2
4
Hexyl acetate standard
5
9.20
9.40
9.60
9.80
10.00
10.20
10.40
Abundance
Retention time
MS of Hexyl acetate standard
6
100
0
100
50
60
70
80
90
100
110
120
130

B

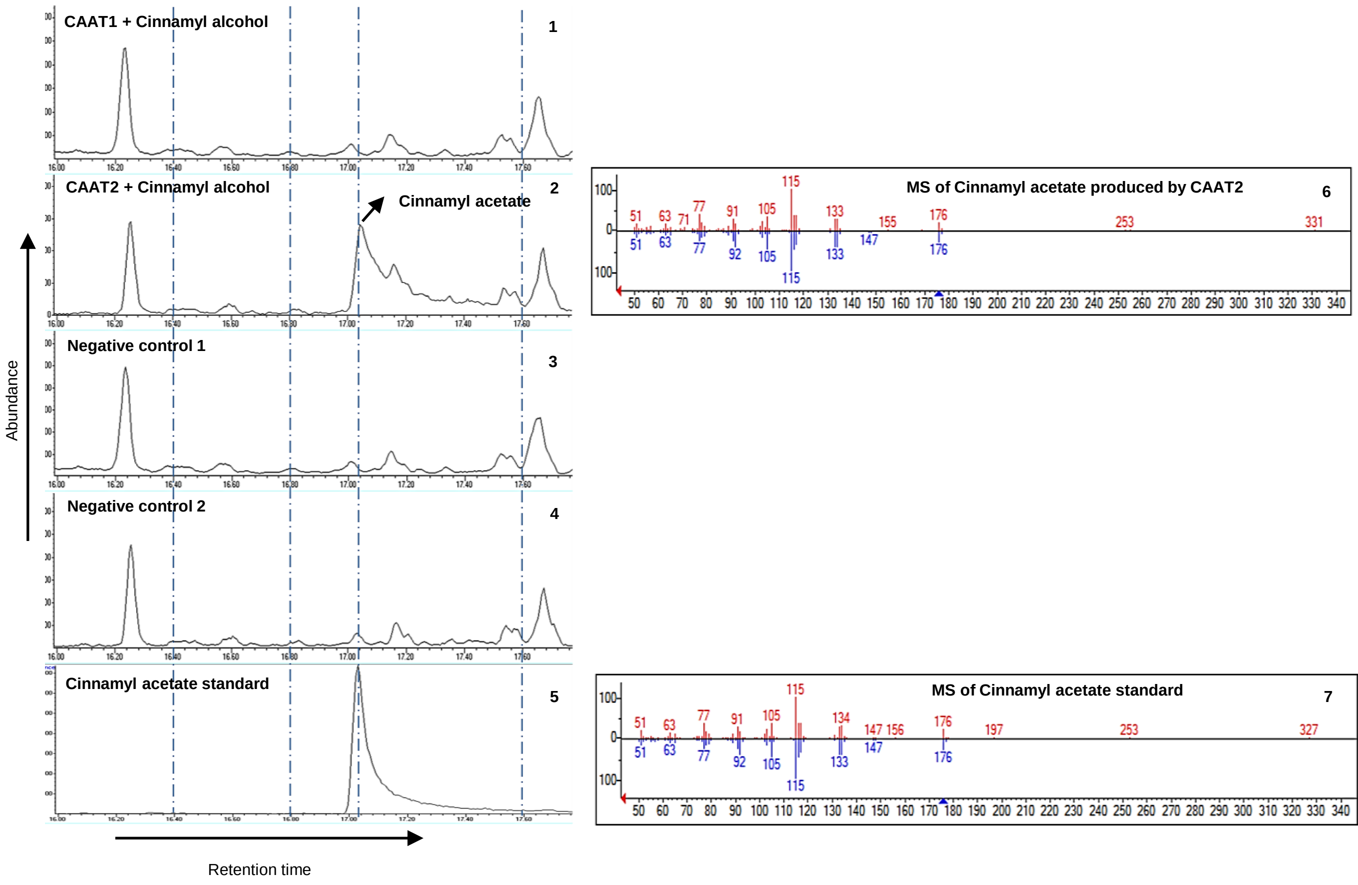
CAAT1 + Cinnamyl alcohol
1
CAAT2 + Cinnamyl alcohol
Cinnamyl acetate
2
Negative control 1
3
Negative control 2
4
Cinnamyl acetate standard
5
Abundance
Retention time
MS of Cinnamyl acetate produced by CAAT2
6
MS of Cinnamyl acetate standard
7

C

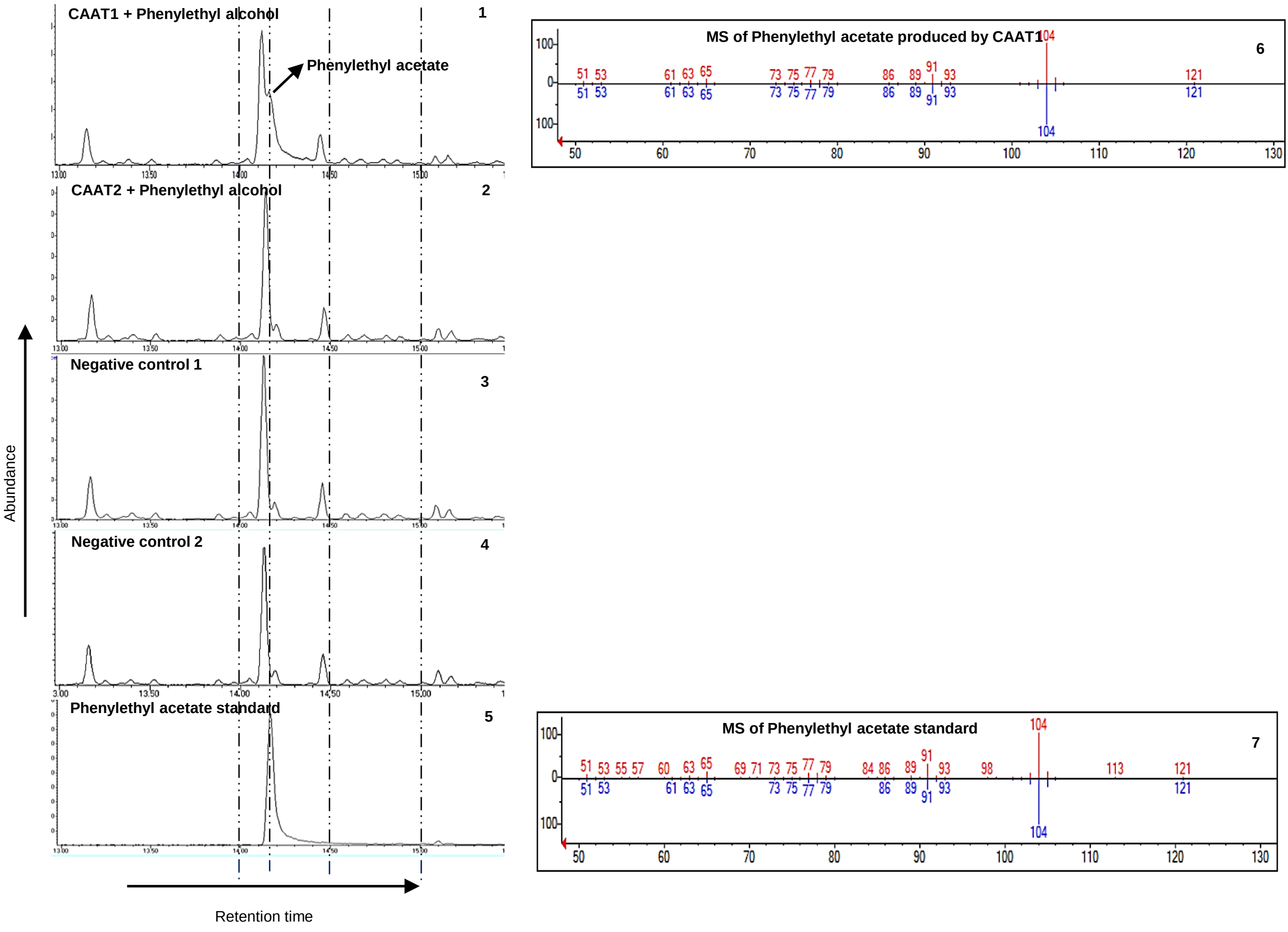
CAAT1 + Phenylethyl alcohol
1
Phenylethyl acetate
CAAT2 + Phenylethyl alcohol
2
Negative control 1
3
Negative control 2
4
Phenylethyl acetate standard
5
Abundance
Retention time
MS of Phenylethyl acetate produced by CAAT1
6
MS of Phenylethyl acetate standard
7

D

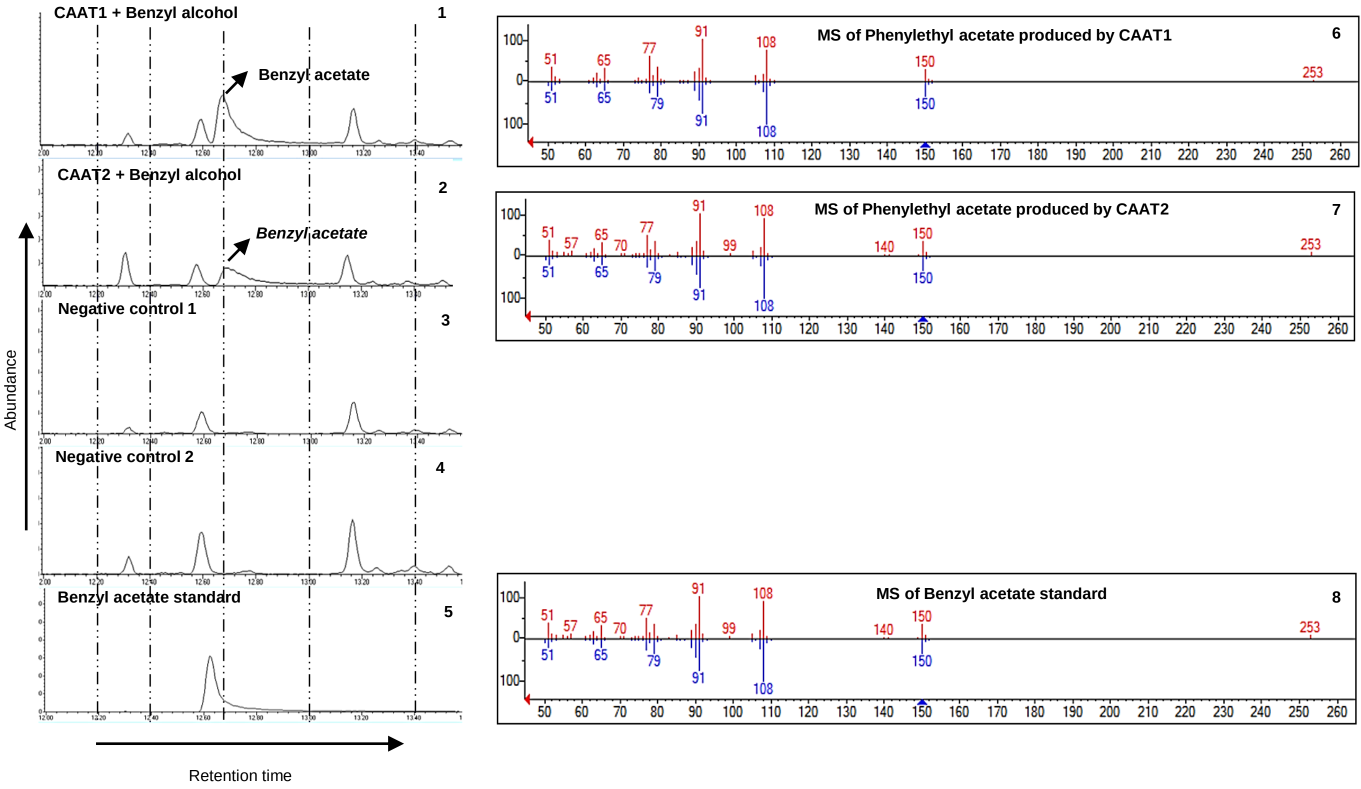
CAAT1 + Benzyl alcohol
1
Benzyl acetate
CAAT2 + Benzyl alcohol
2
Benzyl acetate
Negative control 1
3
Negative control 2
4
Benzyl acetate standard
5
Abundance
Retention time
MS of Phenylethyl acetate produced by CAAT1
6
MS of Phenylethyl acetate produced by CAAT2
7
MS of Benzyl acetate standard
8
51
57
65
70
77
79
91
99
108
140
150
253
100
0
50
60
70
80
90
100
110
120
130
140
150
160
170
180
190
200
210
220
230
240
250
260

Supplementary Figure 4 A: *In planta* functional characterization of ObCAAT1 and ObCAAT2. GC-MS analysis of products formed by ObCAAT1 and ObCAAT2 in-planta in *N.benthamiana* leaves. **A** (1, 2) Hexyl acetate was not observed in hexanol and ObCAAT1 and ObCAAT2 infiltrated *N. benthamiana* respectively (3) Hexyl acetate was not observed in ObCAAT1 and ObCAAT2 infiltrated *N. benthamiana* (4) Hexyl acetate was not observed in hexanol infiltrated *N. benthamiana* (5) Hexyl acetate standard, (6) Mass spectrogram of hexyl acetate standard. **B** (1) Cinnamyl acetate was not observed in cinnamyl alcohol and ObCAAT1 infiltrated *N. benthamiana* leaves, (2) Cinnamyl acetate was observed in cinnamyl alcohol and ObCAAT2 infiltrated *N. benthamiana* leaves (3) Cinnamyl acetate was not observed in ObCAAT1 and ObCAAT2 infiltrated *N. benthamiana*, (4) Cinnamyl acetate was not observed in cinnamyl alcohol infiltrated *N. benthamiana*, (5) Cinnamyl acetate standard, (6) Mass spectrogram of cinnamyl acetate produced by ObCAAT2 in N. benthamiana, (7) Mass spectrogram of cinnamyl acetate standard. **C** (1) Phenylethyl acetate was observed in phenylethyl alcohol and ObCAAT1 infiltrated *N. benthamiana* leaves, (2) Phenylethyl acetate in phenylethyl alcohol and ObCAAT2 infiltrated *N. benthamiana* leaves (3) Phenylethyl acetate was not observed in ObCAAT1 and ObCAAT2 infiltrated *N. benthamiana*, (4) Phenylethyl acetate was not observed in phenylethyl alcohol infiltrated *N. benthamiana*, (5) Phenylethyl acetate standard, (6) Mass spectrogram of phenylethyl acetate produced by ObCAAT1 in N. benthamiana, (7) Mass spectrogram of phenylethyl acetate standard. **D** (1) Benzyl acetate was observed in benzyl alcohol and ObCAAT1 infiltrated *N. benthamiana* leaves, (2) Benzyl acetate was observed in benzyl alcohol and ObCAAT2 infiltrated *N. benthamiana* leaves (3) Benzyl acetate was not observed in ObCAAT1 and ObCAAT2 infiltrated *N. benthamiana*, (4) Benzyl acetate was not observed in benzyl alcohol infiltrated *N. benthamiana*, (5) Benzyl acetate standard, (6) Mass spectrogram of benzyl acetate produced by ObCAAT1 in *N. benthamiana*, (7) Mass spectrogram of benzyl acetate produced by ObCAAT2 in *N. benthamiana*, (8) Mass spectrogram of benzyl acetate standard.

A

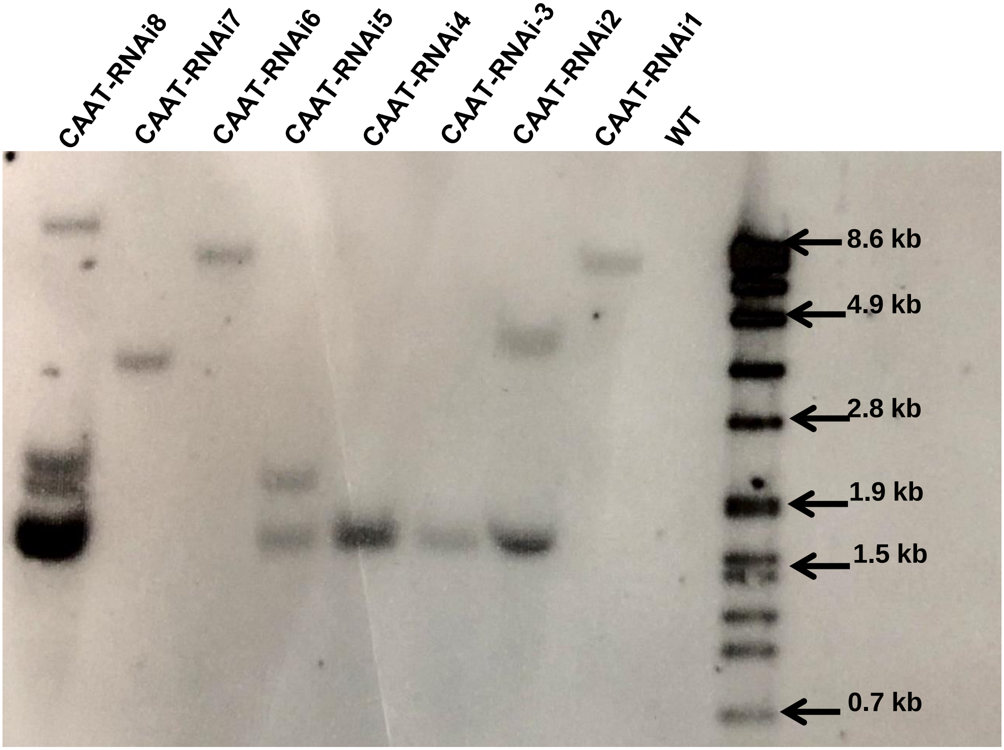

B

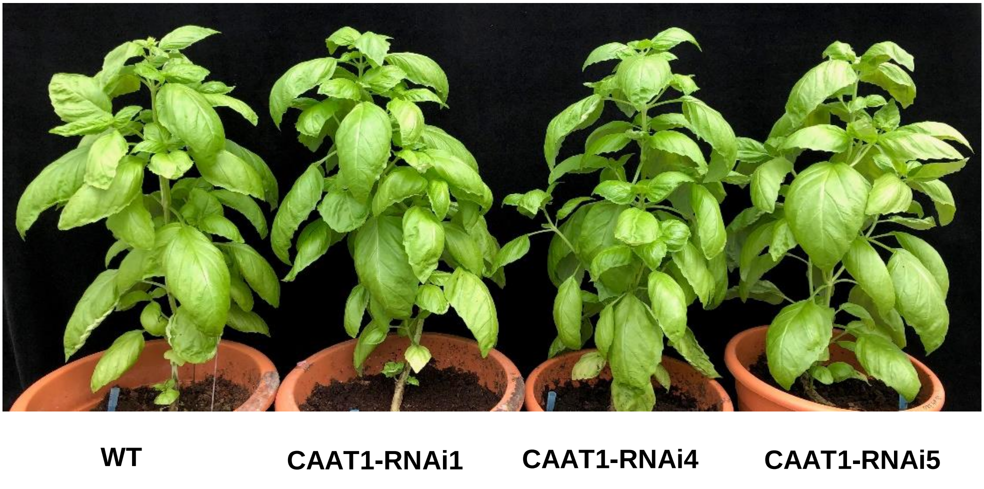

Supplementary Figure 5. Southern blot and phe**notypic analysis of *ObCAAT1*-RNAi sweet basil lines.** (A) Southern blot of *ObCAAT1*-RNAi sweet basil lines showing different T-DNA insertions. 20μg of DNA was digested with *NdeI* enzyme. (B) No phenotypic change was observed in *ObCAAT*- RNAi lines with respect to wild type.

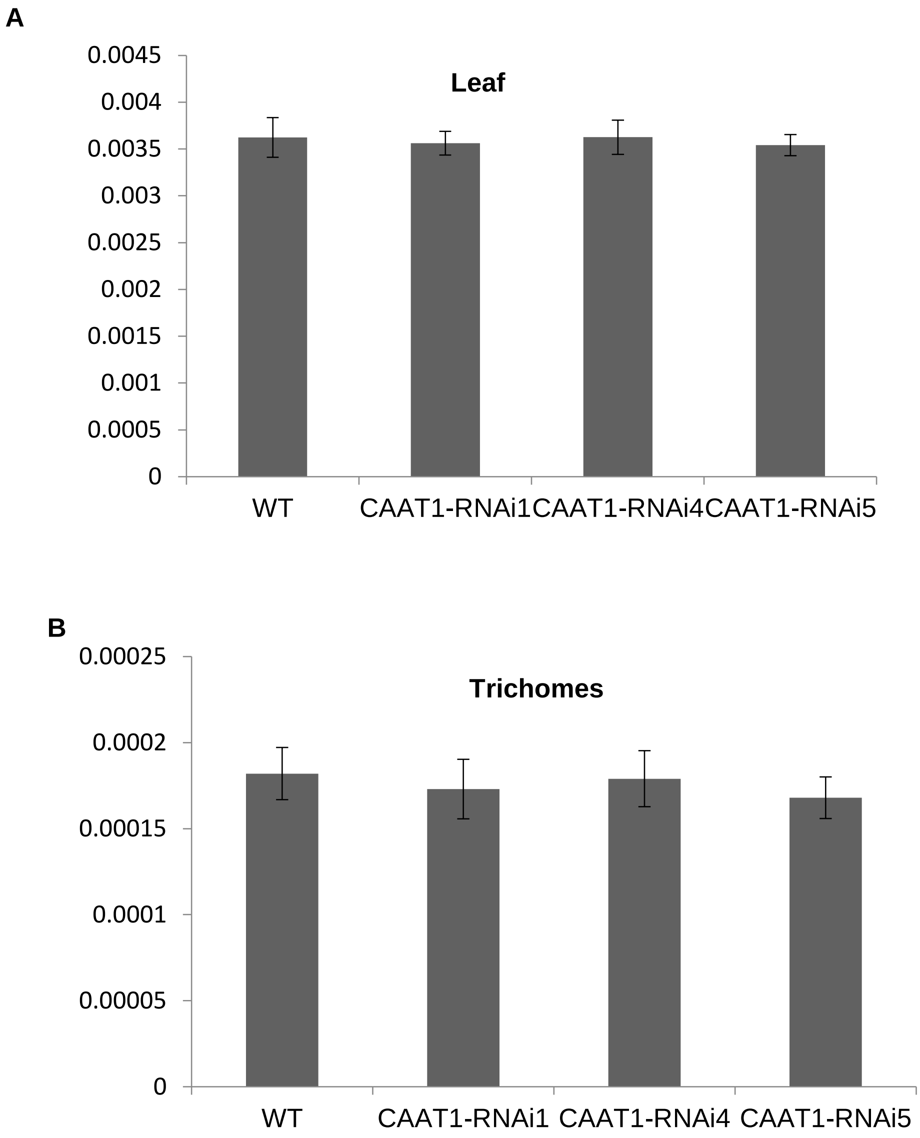

Supplementary Figure 6. Total lignin quantification of *ObCAAT*-RNAi sweet basil lines by thioglycolic acid method. Unaltered lignin content in (A) leaf and (B) trichomes of *ObCAAT1*-RNAi lines when compared to WT. Values are mean ± SE (n = 6).

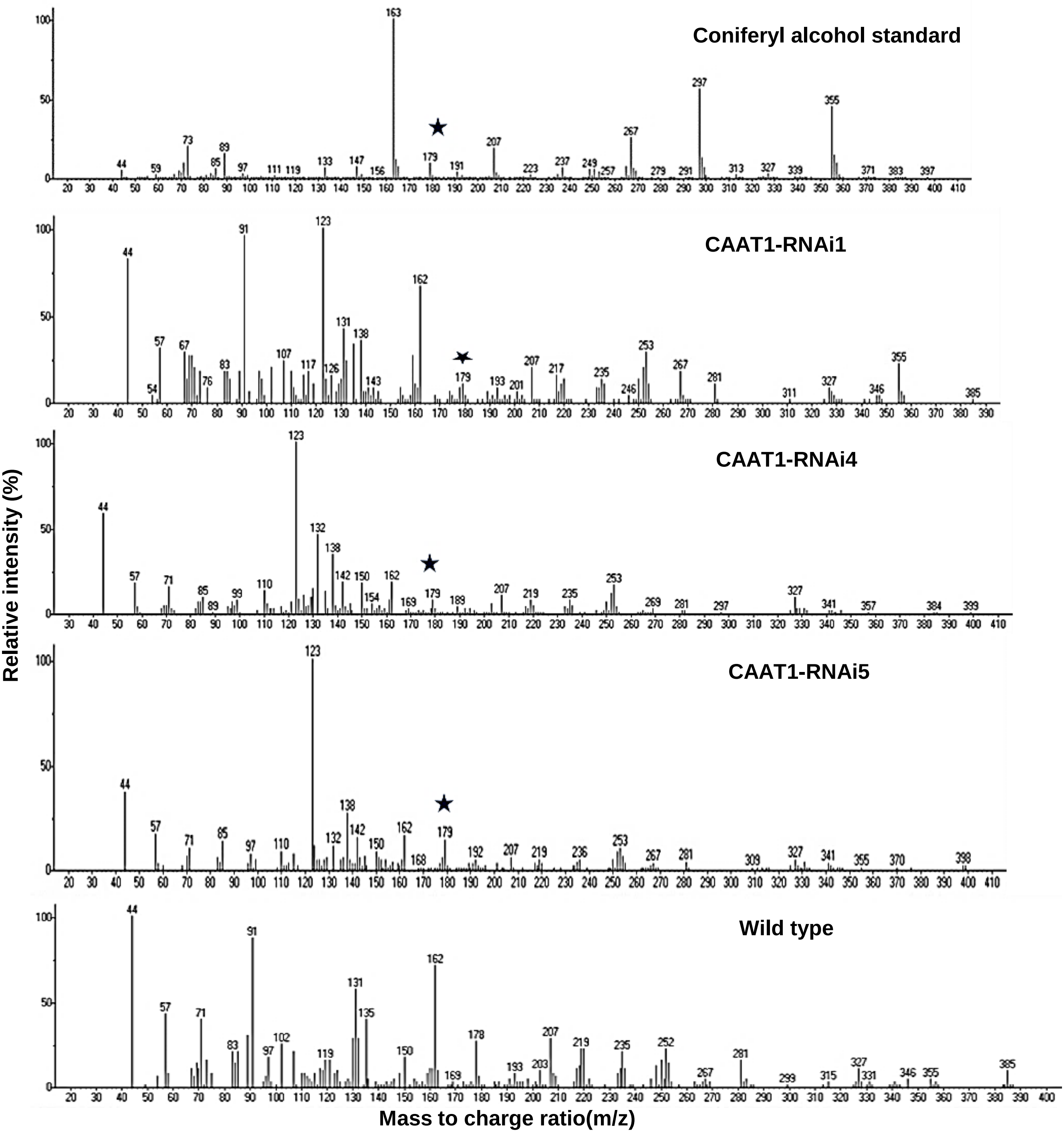

Coniferyl alcohol standard
CAAT1-RNAi1
CAAT1-RNAi4
CAAT1-RNAi5
Wild type
Relative intensity (%)
Mass to charge ratio(m/z)

Supplementary Figure 7. MS spectrum with fragmentation peak at m/z 179 for coniferyl alcohol. Fragmentation peak at m/z 179 for coniferyl alcohol was present in (A) coniferyl alcohol standard, (B) CAAT1-RNAi1, (C) CAAT1-RNAi4, (D) CAAT1-RNAi5 and absent in (E) wild type. (Reference: Predicted GCMS spectrum (non-derivatized) of coniferyl alcohol from Pubchem Database).

Supplementary Table 1. List of primers used in present study

Name	Sequence (5'-3')	Purpose
*ObCAAT1*_F	**CACC**ATGGGCGAAGTAGCAAAAGATGA	Full length primer
*ObCAAT1*_R	TCAAGCCTCCATCAAGAAGTCCTT	Full length primer
*ObCAAT2*_F	**CACC**ATGTATATTTTCCCACCTCCACTCCT	Full length primer
*ObCAAT2*_R	CTAAGGTAGGTTGAAAGAGTGAAATGA	Full length primer
*ObCAAT1*_q_F	AAGATGAGAAGAAATTAGTT	qPCR
*ObCAAT1*_q_R	TCAAAATCGTGGCCTCCTTTGT	qPCR
*ObCAAT2*_q_F	AGGAATATAATCCAGCTGTCGG	qPCR
*ObCAAT2*_q_R	AGGAACAAGTCTGGCATGAACAGA	qPCR
ObEF1α- F	AATGGCAAAAAGCTCGAAGA	qPCR
ObEF1α- R	TCGCAGACATGACAGACACA	qPCR
*ObEGS1*_F	**CACC**ATGGAGGAAAATGGGATGAAAAG	Full length primer
*ObEGS1*_R	TTAAAATGCTGCTGAAGCCGGCGGTGGA	Full length primer
*ObCAAT1_LOC*_F	**CACC**ATGGGCGAAGTAGCAAAAGATGA	Subcellular localization
*ObCAAT1_LOC*_R	AGCCTCCATCAAGAAGTCCTTGTCTT	Subcellular localization
*ObCAAT2_LOC*_F	**CACC**ATGTATATTTTCCCACCTCCACTCCT	Subcellular localization
*ObCAAT2_LOC*_R	AGGTAGGTTGAAAGAGTGAAATGA	Subcellular localization
35S(591)-F	CTCAGAAGACCAAAGGGCTATT	Southern blot probe
35S(-34)-R	TGTTTGTTTTGTTGTGGTATTG	Southern blot probe
*ObCAAT1*_SphI	C<u>GCATGC</u>ACAAAGGAGGCCACGATT	RNAi
*ObCAAT1*_NotI	C<u>GCGGCCGC</u>TTGGTGCGCTCCAGGAAT	RNAi
*ObCAAT1*_NdeI	C<u>CATATG</u>ACAAAGGAGGCCACGATT	RNAi
*ObCAAT1*_XbaI	C<u>TCTAGA</u>TTGGTGCGCTCCAGGAAT	RNAi
